# Exercise Equals the Mobilization of Visceral versus Subcutaneous Adipose Fatty Acid Molecules in Fasted Rats Associated with the Modulation of the AMPK/ATGL/HSL Axis

**DOI:** 10.3390/nu15143095

**Published:** 2023-07-10

**Authors:** Tiziana Zotti, Antonia Giacco, Arianna Cuomo, Luigi Cerulo, Giuseppe Petito, Stefania Iervolino, Rosalba Senese, Federica Cioffi, Pasquale Vito, Gaetano Cardinale, Elena Silvestri, Assunta Lombardi, Maria Moreno, Antonia Lanni, Pieter de Lange

**Affiliations:** 1 Dipartimento di Scienze e Tecnologie, Università degli Studi del Sannio, Via De Sanctis, 82100 Benevento, Italy; tzotti@unisannio.it (T.Z.); agiacco@unisannio.it (A.G.); lcerulo@unisannio.it (L.C.); stefaniaiervolino15@gmail.com (S.I.); federica.cioffi@unisannio.it (F.C.); pasqualevito@gmail.com (P.V.); silves@unisannio.it (E.S.); moreno@unisannio.it (M.M.); 2 Genus Biotech Srls., Università degli Studi del Sannio, Apollosa, 82030 Benevento, Italy; 3 Dipartimento di Scienze e Tecnologie Ambientali, Biologiche e Farmaceutiche, Università degli Studi della Campania “Luigi Vanvitelli”, Via Vivaldi 43, 81130 Caserta, Italy; arianna.cuomo@unicampania.it (A.C.); giuseppe.petito@unicampania.it (G.P.); rosalba.senese@unicampania.it (R.S.); antonia.lanni@unicampania.it (A.L.); 4 Sannio Tech Consortium, s.s. Appia, Apollosa, 82030 Benevento, Italy; gaetano.cardinale@tecnobios.com; 5 Dipartimento di Biologia, Università degli Studi di Napoli “Federico II”, Monte Sant’Angelo, Via Cinthia 4, 80126 Naples, Italy; aslombar@unina.it

**Keywords:** fasting, exercise, fatty acids, lipidomic analysis, visceral white adipose tissue, subcutaneous white adipose tissue, ATGL, HSL, liver, serum

## Abstract

Combining exercise with fasting is known to boost fat mass-loss, but detailed analysis on the consequential mobilization of visceral and subcutaneous WAT-derived fatty acids has not been performed. In this study, a subset of fasted male rats (66 h) was submitted to daily bouts of mild exercise. Subsequently, by using gas chromatography—flame ionization detection, the content of 22 fatty acids (FA) in visceral (v) versus subcutaneous (sc) white adipose tissue (WAT) depots was compared to those found in response to the separate events. Findings were related to those obtained in serum and liver samples, the latter taking up FA to increase gluconeogenesis and ketogenesis. Each separate intervention reduced scWAT FA content, associated with increased levels of adipose triglyceride lipase (ATGL) protein despite unaltered AMP-activated protein kinase (AMPK) Thr172 phosphorylation, known to induce ATGL expression. The mobility of FAs from vWAT during fasting was absent with the exception of the MUFA 16:1 n-7 and only induced by combining fasting with exercise which was accompanied with reduced hormone sensitive lipase (HSL) Ser563 and increased Ser565 phosphorylation, whereas ATGL protein levels were elevated during fasting in association with the persistently increased phosphorylation of AMPK at Thr172 both during fasting and in response to the combined intervention. As expected, liver FA content increased during fasting, and was not further affected by exercise, despite additional FA release from vWAT in this condition, underlining increased hepatic FA metabolism. Both fasting and its combination with exercise showed preferential hepatic metabolism of the prominent saturated FAs C:16 and C:18 compared to the unsaturated FAs 18:1 n-9 and 18:2 n-6:1. In conclusion, depot-specific differences in WAT fatty acid molecule release during fasting, irrelevant to their degree of saturation or chain length, are mitigated when combined with exercise, to provide fuel to surrounding organs such as the liver which is correlated with increased ATGL/ HSL ratios, involving AMPK only in vWAT.

## 1. Introduction

Basic research on animal models as well as studies on humans have revealed that combined interventions aiming to achieve fat mass loss by combining energy restriction with exercise are beneficial [1]. The accumulation of visceral white adipose tissue (vWAT) is a risk factor for metabolic syndrome and insulin resistance irrespective of BMI [2,3]. Combining exercise with energy restriction has been shown to be particularly effective in reducing both total and visceral fat mass and HBA1c in obese women [4]. A literature survey of 89 studies has revealed that weight-loss interventions including dietary interventions with exercise in humans do not preferentially target vWAT, and that vWAT mass loss is tightly linked to scWAT mass loss [5]. In humans, fat mass loss/gain is not predictive of fatty acid (FA) release/gain [6]. To understand how adipose tissue influences metabolic responses, detailed tissue-specific analysis of fatty acid FA release is warranted, which is relatively more feasible in animal models. In this light, it has been shown in rats that fasting does not induce vWAT fatty acid mobilization and content within 24 h, but only after very long timespans (7 and 10 days) [7]. The content of FAs from lipid classes has been measured previously in rat scWAT, vWAT and liver in response to 8 weeks of treadmill exercise [8], and in livers of male rats in response to time restricted high-fat feeding [9]. It is not known to what extent vWAT contributes to the supply of lipids to the liver with respect to scWAT during prolonged periods of fasting within the timespan considered scientifically acceptable for rats according to guidelines by Boston University (https://www.bu.edu/researchsupport/compliance/animal-care/working-with-animals/foodregulation-and-restriction-in-rodents, accessed on 1 December 2020). In addition, it is at present unknown whether these events can be modulated by short bouts of mild endurance exercise during the fasting period. Adipose FA mobilization is the result of the cleavage of triglycerides by Adipose triglyceride lipase (ATGL) and diglycerides by hormone sensitive lipase (HSL) [10], which is particularly impacted by fasting [11]. Despite decreased hepatic lipogenesis during prolonged fasting and caloric restriction [12,13], lipids accumulate in the liver. This has been shown to be the result of competition between mitochondrial and peroxisomal beta oxidation [14], and the increased supply of non-esterified fatty acids released from white adipose tissue [15]. The accumulated lipids serve to stimulate gluconeogenesis and ketogenesis [12,16]. In this study, using gas chromatography- flame ionization detection (GC-FID) and Western Immunoblot analysis we aimed to assess how mild endurance exercise influenced the relationship between the mobilization of a selection of 22 fatty acids from vWAT and scWAT, and their subsequent levels observed in liver. We related our findings to changes in the ratios of HSL versus ATGL.

## 2. Materials and Methods

### 2.1. Materials

The primary antibodies against phospho-HSL (Ser^563^ and Ser^565^), pan-HSL, ATGL, Phospo-AMPK (Thr^172^), pan-AMPK, were obtained from Cell Signaling Technology (Beverly, MA, USA). The β-actin antibody was from Bioss Antibodies Inc. (Woburn, MA, USA). The appropriate horseradish peroxidase-conjugated secondary antibodies were purchased from Abcam (Cambridge, UK). The commercial mixture of 22 fatty acid methyl ester standard was from Nu-Chek-Prep (Elysian, MN, USA).

### 2.2. Animal Handling

Rats (strain: Wistar, sex: male, n = 16, age: 12 weeks, weight: approximately 300 g) were housed separately at thermoneutrality (28 °C), having ad libitum access to water and chow [Fatty acid content (mg/kg): palmitate (16:0) 4387; palmitoleate (16:1) 202; stearic (18:0) 675; oleic (18:1) 5046; linoleic (18:2) 12,335; linolenic (18:3) 1169. Total metabolizable percentage of energy: carbohydrates 60.4; proteins 29; fat 10.6; 15.88 KJ gross energy/g (Muscedola s.r.l., Milan, Italy)]. During the 3-week-acclimation period, rats were gradually familiarized with a treadmill (Panlab, Harvard Apparatus, Holliston, MA, USA). Four experimental groups were used, each consisting of four rats. The first underwent ad libitum feeding (C group), the second ad libitum feeding with exercise (E group), the third 66 h fasting (F group), and the fourth 66 h fasting and exercise (FE group). The exercised groups (E and FE) were engaged in five treadmill runs (twice daily, 30 min, 15 m/min, 0° inclination). During the runs the temperature inside the treadmill cover was set at 25 °C and did not exceed 28 °C throughout. Animals were placed back in their cages at 28 °C 15 min after finishing the run. A timeline of the experimental setup and body weight, food intake, and metabolic parameters were published elsewhere [12]. Sacrifice occurred 4 h after completion of the last exercise bout. Next, serum was collected and liver and visceral (combined mesenteric, gonadic, perirenal, retroperitoneal) versus subcutaneous (flank) white adipose tissue (WAT) depots were quickly removed, immediately frozen in liquid nitrogen, and stored at −80 °C. Animal experiments complied with the ARRIVE guidelines and were carried out following the EU Directive 2010/63/EU for animal experiments. Each protocol was approved by the Ethics of Animal Experiments Committee of the University of Campania “Luigi Vanvitelli” and the Italian Health Ministry (authorization 704/2016 PR, article 31 legislative decree 26/2014).

### 2.3. Lipid Analysis

Fatty acid extraction and derivatization was performed according to a previously described method [17]. Briefly, pieces of liquid nitrogen-frozen tissue were coarsely fragmented and homogenized for about 1 min in a 20-mL glass vial containing 2 mL of 12% *w*/*v*, 1.5 M boron trifluoride-methanol (BF3-MeOH, purchased from Acros Organics, Geel, Belgium). Then, vials were sealed and kept at 100 °C for 1 h, to allow FA transesterification. After cooling down the vials, 2 mL of n-Hexane (Carlo Erba Reagents Srl., Val de Reuil, France) were added to the mixture and vortexed, resulting in the formation of an upper n-Hexane transparent layer containing fatty acid methyl esters (FAMEs). FAMEs were then extracted, put into a 2 mL glass GC-vial and air-dried. Later, FAMEs were resuspended in 400 μL of n-Hexane and injected in the GC-FID for analysis.

### 2.4. GC-FID Analysis of FAMEs

The determination of FAMEs was performed on a GC-2010 gas chromatograph (Shimadzu, Kyoto, Japan), equipped with an SP^®^-2560 capillary GC column (L × I.D. 100 m × 0.25 mm × 0.2 μm) and a flame ionization detector as described in previous reports [18,19]. FAMEs were identified by using the retention times of 22 known FAMEs in a standard mixture (Nu-Chek-Prep, Elysian, MN, USA) and quantitative determination of individual FAME was carried out by comparing peak area values with calibration curves prepared using five batches of FAME standards. The association between the instrument response (peak area) and the concentration of individual FAME in the analyte was obtained by regression analysis as in [19]. Data were analyzed by using Shimadzu system GC Solutions software (Shimadzu Europa GmbH, Duisburg, Germany), designed for this system. The amounts of FAME are expressed in nanograms per mg of processed tissue or, in the case of serum, nanograms per mL.

### 2.5. Western Immunoblot Analysis

Protein and phosphoprotein levels were determined by immunoblotting using total lysates from subcutaneous or visceral white adipose tissue by employing the above-described specific antibodies. β actin was used as a loading control.

### 2.6. Statistical Analysis

The minimal number of animals required for the study was determined by a g-power test [G*Power software version 3.1.9.2 from the Heinrich Heine University of Dusseldorf (http://www.gpower.hhu.de, accessed on 1 December 2018)], as reported previously [12]. All animals were analyzed to produce the data. We adopted two-way ANOVA with fixed effects to test main and interaction effects of the two experimental interventions: Presence/Absence of Exercise and Presence/Absence of Fasting in the mean levels of Lipids. Specifically, we tested for the following three alternative hypotheses of main and interaction effects: (i) there is a difference between the Presence and Absence of Exercise; (ii) there is a difference between the Presence and Absence of Fasting; and (iii) the effect of Exercise depends on the Presence or Absence of Fasting. In addition, we tested whether the interventions caused differences in the proteins related to lipase activity in the tissues. Post-hoc Tukey-Kramer tests were performed to determine statistical significance between the means of each intervention, presented with their respective standard deviations throughout. Statistical analyses were performed with R [20] and Prism 8.0 (Graphpad, San Diego, CA, USA). Differences were considered significant at *p* < 0.05.

## 3. Results

**Figure 1:**
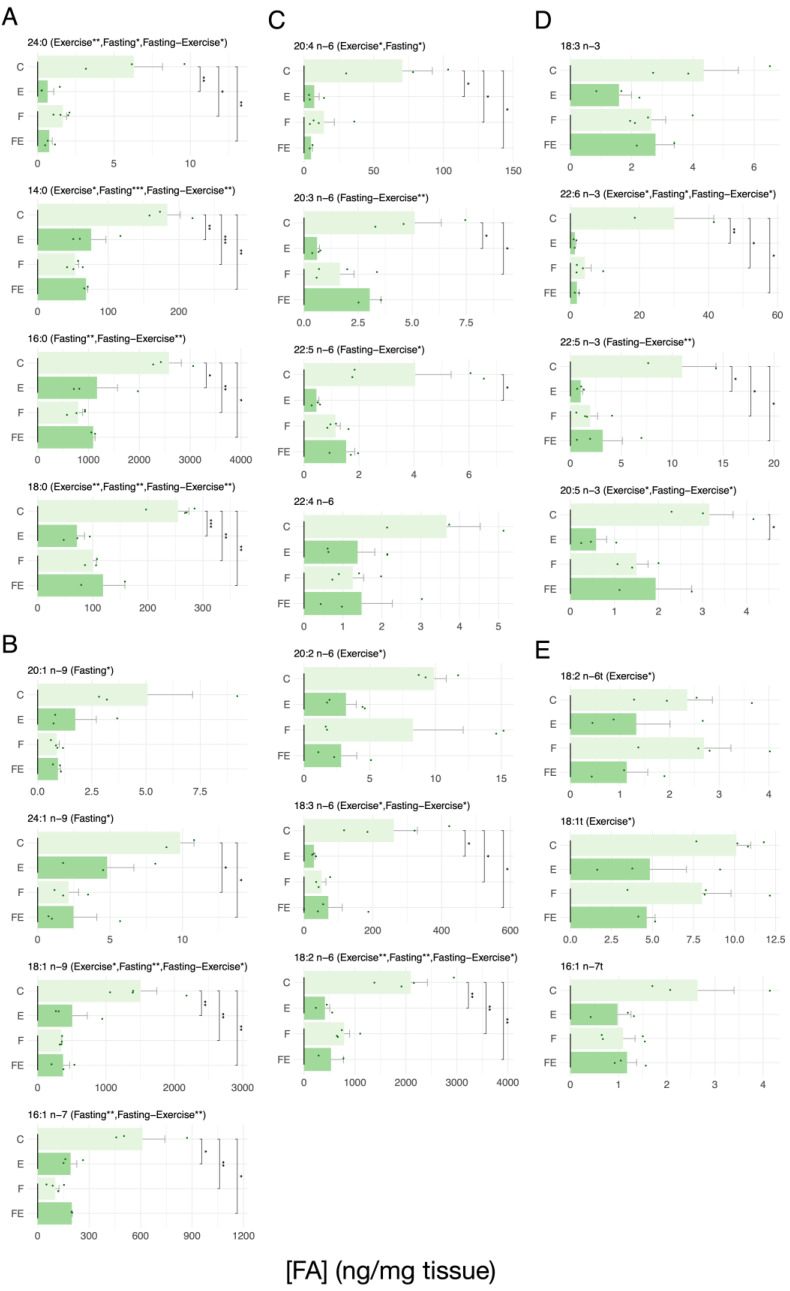
Absolute levels of each measured FA in scWAT in response to each intervention. (**A**) SFA, (**B**) MUFA, (**C**) n6 PUFA, (**D**) n3 PUFA, (**E**) TFA. (N = 4) For data presentation and statistical analysis see Section 2. Symbols above the histograms represent different ranges of *p*-values. * *p* < 0.05, ** *p* < 0.01, *** *p* < 0.001.

**Figure 2:**
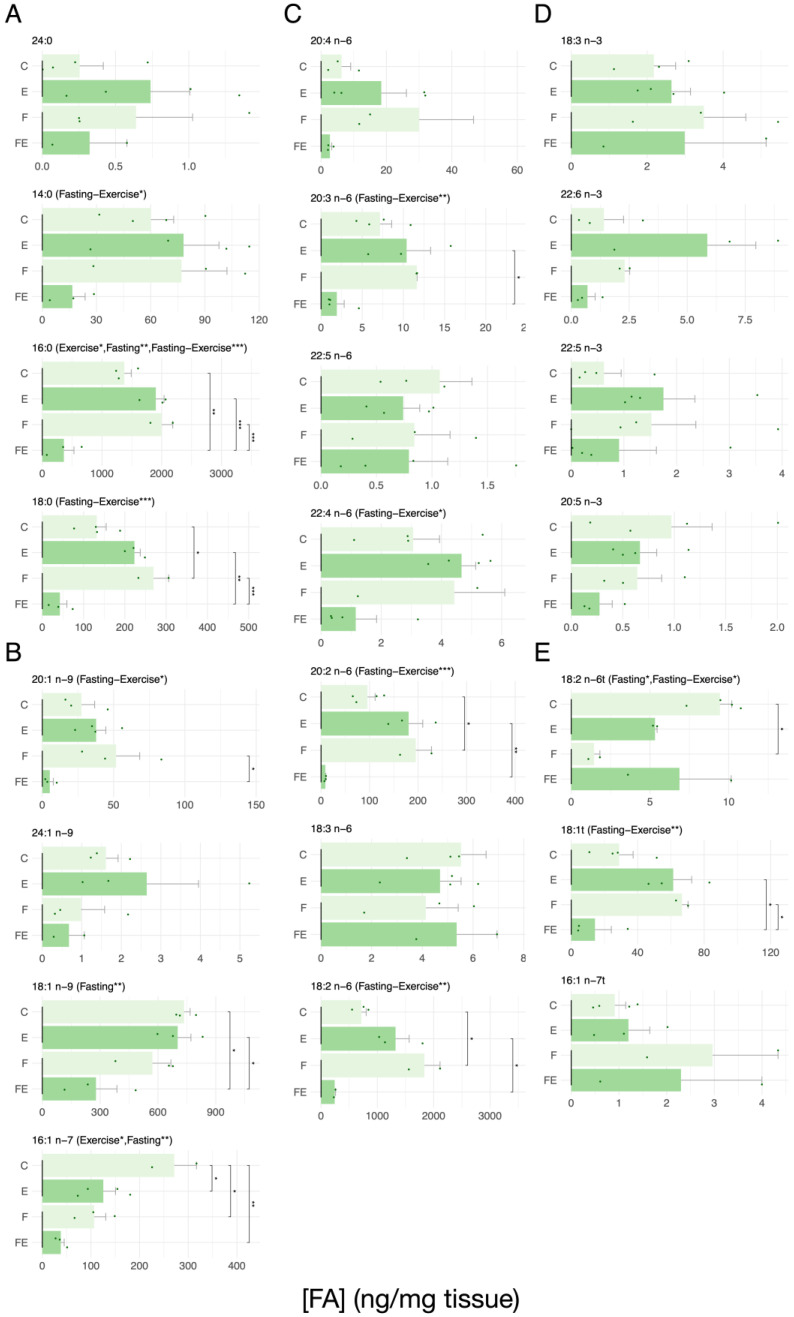
Absolute levels of each measured FA in vWAT in response to each intervention. (**A**) SFA, (**B**) MUFA, (**C**) n6 PUFA, (**D**) n3 PUFA, (**E**) TFA. (N = 4) For data presentation and statistical analysis see Section 2. Symbols above the histograms represent different ranges of *p*-values. * *p* < 0.05, ** *p* < 0.01, *** *p* < 0.001.

**Figure 3:**
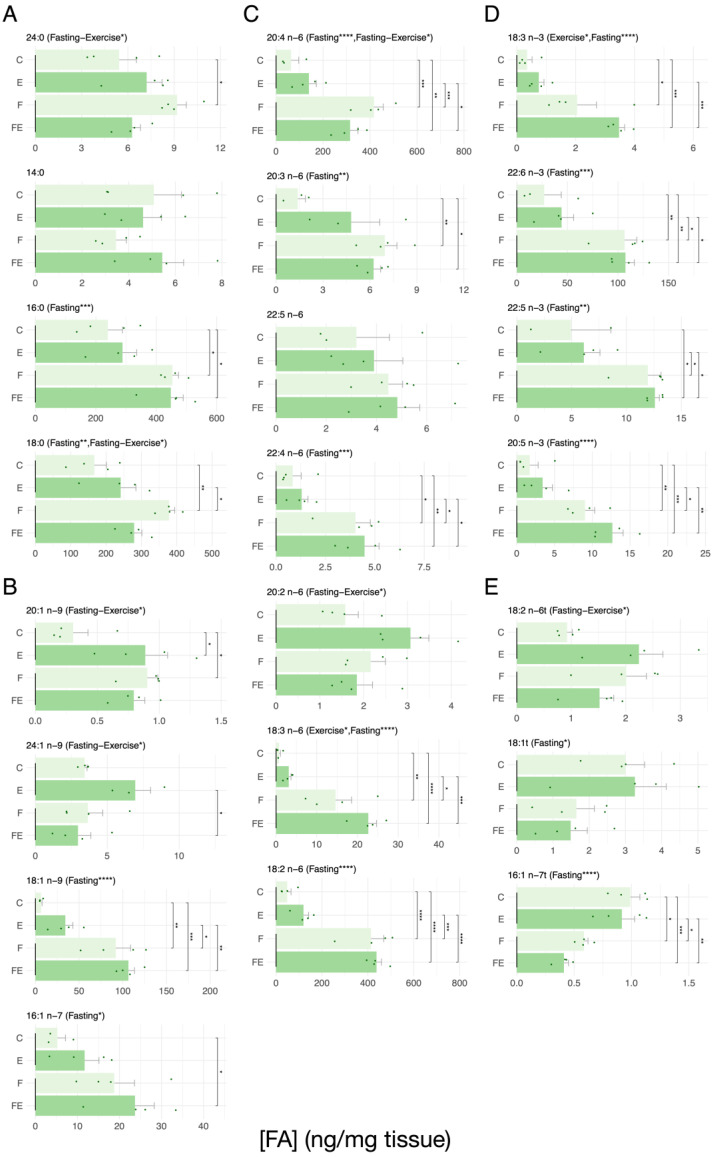
Absolute levels of each measured FA in liver in response to each intervention. (**A**) SFA, (**B**) MUFA, (**C**) n6 PUFA, (**D**) n3 PUFA, (**E**) TFA. (N = 4) For data presentation and statistical analysis see Section 2. Symbols above the histograms represent different ranges of *p*-values. * *p* < 0.05, ** *p* < 0.01, *** *p* < 0.001, **** *p* < 0.0001.

**Figure 4:**
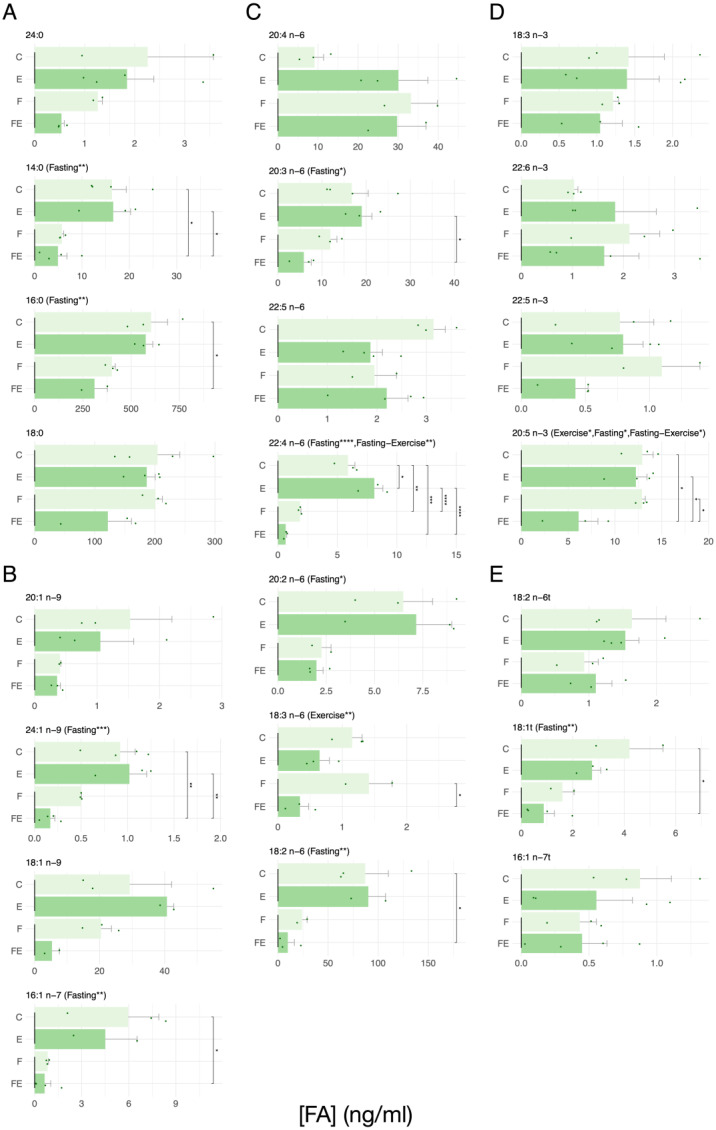
Absolute levels of each measured FA in serum in response to each intervention. (**A**) SFA, (**B**) MUFA, (**C**) n6 PUFA, (**D**) n3 PUFA, (**E**) TFA. (N = 4) For data presentation and statistical analysis see Section 2. Symbols above the histograms represent different ranges of *p*-values. * *p* < 0.05, ** *p* < 0.01, *** *p* < 0.001, **** *p* < 0.0001.

With respect to controls (C), we observed that mild exercise (E) suffices to induce the mobilization of the majority of FAs from scWAT, and so does a 66 h fasting period (F), with no change with respect to F in the response to exercise (FE condition) (Figure 1). This was true for all SFAs (24:0, 14:0, 16:0, and 18:0 (Figure 1A)). Similar mobilization efficiencies were observed for the more abundant MUFAs, 18:1 n-9, 16:1 n-7 (Figure 1B). The less abundant MUFA 24:1n-9 showed significant mobilization in the F and FE condition, not in E alone. The MUFA 20:1n-9 did not show significant mobilization. Consistently, the abundant n6 PUFAs 20:4 n-6, 18.3 n-6, and 18:2 n-6 showed increased mobilization in F, E, and FE (Figure 1C). The lesser abundant n6 PUFAs 22:5 n-6 and 20:3 n-6 showed significant mobilization in E, and E and F, respectively. No significant effects were observed for the lesser abundant 22:4 n-6 and 20:2 n-6. The n3 PUFAs 22:6 n3 and 22:5 n3 displayed a similar mobilization in E, F, and FE (Figure 1D), whereas the lesser abundant n3 PUFA 20:5 n-3 showed significant mobilization only in E and 18:3 n-3 and did not vary significantly. With regard to the very lowly abundant TFAs, no significant differences in mobilization were observed upon each intervention (Figure 1E). In order to evaluate the modulatory effect of the combined action of exercise and fasting, we also tested the significance of the interaction between the two interventions nominated as exercise and fasting. Levels of significance of the interactions are indicated above the horizontally displayed histograms in Figure 1, Figure 2, Figure 3 and Figure 4. Interestingly, we found that in scWAT the interaction between exercise and fasting was significant for all SFAs (Figure 1A) albeit that the direction of the effect of fasting was inverted by exercise in 18:0 (Figure 1A). The same was true for the most abundant MUFAs 18:1 n-9 and 16:1 n-7, the latter showing an weak inversion of the direction of the effect of fasting by exercise (Figure 1B), for the n6 PUFAs 20:3 n-6, 22:5 n-6, 18:3 n-6, 18:2 n-6 and for all the n3 PUFAs with the exception of 18:3 n-3 the direction of the effect of fasting was inverted by exercise (Figure 1C, and D, respectively). Regarding to TFAs, we could not observe significant interactions between the two interventions (Figure 1E).

In contrast, vWAT resulted to be less prone to FA mobilization than scWAT (Figure 2). In general, only FE resulted in increased mobilization. This was true for the SFA 16:0 and 18:0, with respect to C, E, and F, and E and F, respectively (Figure 2A). There was an FE induced mobilization ratio of 14:0 but this did not reach significance (*p* = 0.35), with the lesser abundant 24:0 showing no change. A ratio of 18:0 was also significantly mobilized in F with respect to C. Mobilization of the MUFA 18:1 n-9 in FE reached significance with respect to C and E (Figure 2B). With respect to F, the MUFA 20:1 n-9 showed a significantly higher mobilization in FE. One interesting exception is the MUFA 16:1 n-7, in which mobilization equaled that observed in in scWAT, being increasingly mobilized in each condition (Figure 2B). No changes in response to the interventions were observed for the less abundant 24:1 n-9 (Figure 2B). The three abundant n6 PUFAs 20:3 n-6, 20:2 n-6, and 18:2 n-6 showed increased FA mobilization in FE with respect to F and E alone (Figure 2C). Significance was not reached for 20:4 n-6 (*p* = 0.245 with respect to F, and *p* = 0.611 with respect to E), and the lower abundant n6 PUFAs 22:5 n-6, 22:4 n-6, and 18:3 n-6 showed no changes. Interestingly, 20:2 n-6 and 18:2 n-6 significantly accumulated in F with respect to C. The n3 PUFAs were lowly abundant and showed no changes in response to the interventions in this tissue (Figure 2D). As expected, the F condition reduced the abundance of the TFA 18:2 n-6t, and FE increased mobilization of 18:1t with respect to F and E. (Figure 2E). Regarding each measured FFA, the interactions between the effect of fasting and exercise on their levels were also assessed (significance of the interactions is indicated above each histogram in Figure 1, Figure 2, Figure 3 and Figure 4). The effect of exercise on the mean level of SFAs 14:0, 16:0, and 18:0 depends on fasting. Specifically, in the presence of fasting, exercise causes a drop in lipid levels which is significantly higher than in the absence of fasting (Figure 2A). The same is true for 20:1n-9 (Figure 1B) 20:3 n-6, 20:2 n-6,18:2 n-6, with a weaker effect on 22:4 n-6 (Figure 2C), and 18:1t, with the effect of fasting being inverted by exercise and vice versa in 18:2 n-6t (Figure 2E).

It is worth noting that the increased mobility of fatty acids observed in scWAT and vWAT is accompanied by a statistically significant hepatic accumulation of fatty acids compared to the control condition C (see Figure 3). With regard to the SFA, this held true for 16:0, whereas 18:0 increased in F, with a tendency reached in FE (*p* = 0.094) (Figure 3A). F also significantly increased the less abundant SFA 24:0. Interestingly, the levels of 14:0 were low and unaffected (Figure 3A), although this latter FA is abundantly present in WAT and is significantly mobilized in scWAT (Figure 1A). The more abundant MUFA 18:1 n-9 significantly increased both in F and FE, whereas 16:1n-7 increased in FE (Figure 3B), with a tendency in F (*p* = 0.175). Of the lower abundant MUFAs, 20:1 n-9 was significantly increased in E and F (Figure 3B), but in FE significance was not reached (*p* = 0.069). In apparent contrast, this FA was observed to be abundant in vWAT and mobilized in FE with respect to F (Figure 2B). The FA 24:1 n-9 decreased in FE with respect to F, and this FA was observed to also be lowly abundant in sc and vWAT (Figure 1B and Figure 2B). Each of the three measured abundant n6 PUFAs 20:4 n-6, 18:3 n-6, and 18:2 n-6 as well as the lower abundant 20:3 n-6 and 22:4 n-6 significantly increased in F and FE (Figure 3C). The less abundant 22:5 n-6 and 20:2 n-6 showed no change, although the latter is abundant in vWAT and is mobilized in FE (Figure 2C). Each measured n3 PUFA follows an identical pattern, namely an increase in the F and FE condition (Figure 3D). In case of the less abundant 22:5 n-3, the increase in F did not reach significance but showed a strong tendency (*p* = 0.052), whereas with respect to E the increase in the F condition reached significance (Figure 3D). Regarding the TFAs, being of low abundance in the liver, 16:1 n-7t is significantly reduced in F and FE (Figure 3E). The exercise condition (E), although significantly decreasing the FA pools in scWAT (Figure 1), results only in a tendential increase in the measured FAs in liver (Figure 3), with the exception of the less abundant 20:1 n-9 (Figure 3B). Regarding the interactions between the effect of fasting and exercise on FA levels, altogether, these data indicate that the accumulation of FAs in liver is mainly driven by fasting. Nevertheless, we found a significant interaction between exercise and fasting for the SFA 18:0, the effect of fasting was inhibited by exercise (Figure 3A), the same was true for the MUFAs 20:1 n-9 and 24:1 n-9, in which in presence of fasting, the stimulatory effect of exercise is normalized to C values (Figure 3B), for the n6 PUFAs 20:4 n-6 the effect of fasting was inhibited by exercise and for 20:2 n-6, and the TFA 18:2 n-6t the effect of fasting was inhibited by exercise (Figure 3C,E). We did not observe any interaction for the analyzed n-3 PUFAs (Figure 3D).

We also measured the presence of the FAs in serum, and we found that, apart from one exception, the only condition in which several of these were significantly reduced is FE (Figure 4). These FAs include the SFAs 14:0 and 16:0 (Figure 4A), the MUFAs 24:1 n-9 and 16:1 n-7 (Figure 4B), and the n6 PUFA 18:2 n-6 (Figure 4C). Each of these FAs decreased with respect to C. Further, the n6 PUFA 20:3 n-6 decreased with respect to E, 18:3 n-6 decreased with respect to F, and 22:4 n-6, apart from decreasing in FE, increased in E and decreased in F (Figure 4D). One n3 PUFA that decreased in FE with respect to C is 20:5 n-3 (Figure 4D), and the same was true for the TFA 18:1t (Figure 4E). Regarding the interactions between the effect of fasting and exercise on FA levels, the interaction between exercise and fasting was significant in serum with the effects resulting in the same direction for the n6 PUFA 22:4 n-6 (Figure 4C) and in case of the n3 PUFA 20:5 n-3 the effect of fasting and exercise was synergistic. (Figure 4D).

**Figure 5:**
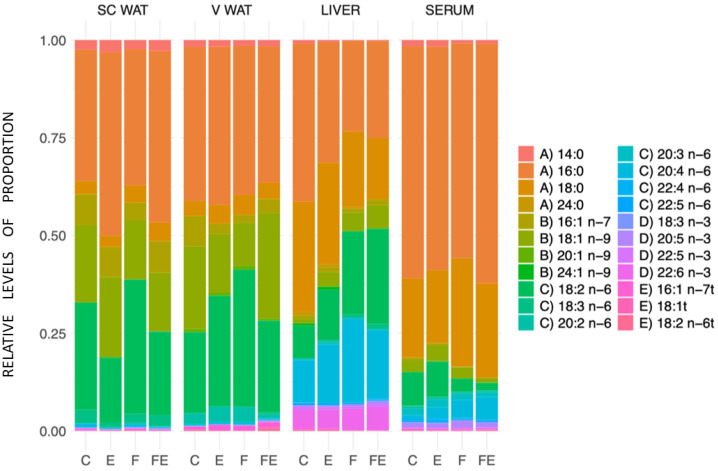
Proportions of the measured FAs in the tissues in response to each intervention. (N = 4) A: SFA, B: MUFA, C: n6 PUFA, D: n3 PUFA, E: TFA.

Subsequently, we asked whether the observed changes in mobility observed in scWAT and vWAT affected the proportions of each measured FA. An overview of the results is presented in Figure 5. We observed no significant change in the proportions of each measured FA in response to each intervention in both tissues (Figures S1 and S2 for scWAT and vWAT, respectively). This implies that WAT exhibits no preferential mobilization of FA related to features such as the degree of saturation or chain length. In the liver, with respect to C, proportions between FAs changed in F, and in FE, the proportion of 16:0 and of 18:0 decreased. There was a compensatory increase in the proportion of 18:1 n-9 and 18:2 n-6 in these conditions, and an increase of 20:4 n-6 in F (Figure S3). In serum, no significant changes in the proportions were observed (Figure S4).

**Figure 6:**
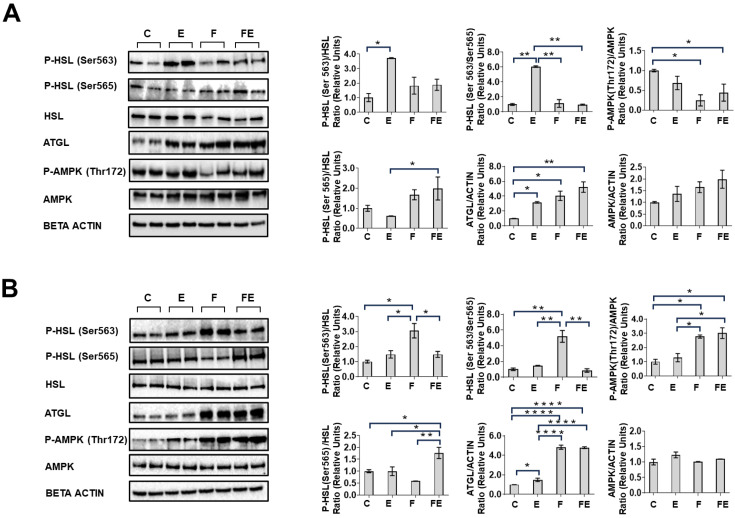
Western immunoblot analysis of HSL, ATGL, and AMPK in representative samples in response to C, E, F, and FE in scWAT (**A**) and vWAT (**B**) (N = 4) with quantified data presented in the histograms. For data presentation and statistical analysis see Section 2. * *p* < 0.05, ** *p* < 0.01, **** *p* < 0.0001.

Next, we performed Western immunoblot analysis to assess whether the observed differences in mobility between scWAT and vWAT in response to each intervention could be related to changes in hormone sensitive lipase (HSL) phosphorylation and/or adipose triglyceride lipase (ATGL) protein levels. In scWAT, with respect to C, E induced a significant 3.7-fold increase in HSL Ser^563^ phosphorylation, and a concomitant significant decrease with respect to the FE of HSL Ser^565^ phosphorylation to 0.3-fold the levels of FE (Figure 6A). As a consequence, the HSL Ser^563^/Ser^565^ ratio in the E condition significantly increased by 6.0-fold with respect to C, E, and FE (Figure 6A). In F and FE, AMPK Thr^172^ phosphorylation was reduced with respect to C (down to 0.2 and 0.4-fold, respectively), while AMPK protein levels increased in response to the interventions, not reaching statistical significance (Figure 6A). ATGL protein levels significantly increased in E, F, and FE (by 3.2, 4.2, and 5.2-fold with respect to C, respectively) (Figure 6A). In vWAT, with respect to C, HSL Ser^563^ phosphorylation was increased by 3.0-fold in F, whereas Ser^565^ phosphorylation significantly increased in FE by 1.8-fold with respect to C, and E, and more significantly so with respect to F (3.0-fold) (Figure 6B). As a consequence, the HSL Ser^563^/Ser^565^ ratio in the F condition significantly increased by 5.2-fold with respect to C, E, and FE (Figure 6B). AMPK Thr^172^ phosphorylation with respect to C increased by 2.7- and 3.0-fold in F and FE, and ATGL protein with respect to C increased by 1.5-fold in E, and by 4.8-fold in both F and FE, respectively (Figure 6B).

## 4. Discussion

This study has revealed that in male rats all investigated FAs (with exception of 16:1 n-7) are more readily mobilized from scWAT when compared to vWAT under the conditions of increased energy demand, irrespective of their degree of saturation, and that this process may involve tissue-specific interplay between ATGL and HSL. An important observation of this study is that exercise can unlock the release of FAs from vWAT during fasting. This is consistent with studies in humans in which combined energy restriction and exercise interventions reduce abdominal fat [4,21]. In our animal model, we did not observe altered proportions of FA in WAT upon each intervention. Previous studies have shown that the fatty acid composition of WAT is affected by long-term energy depletion in vWAT (7–10 days) [7] and that FAs undergo selective mobilization in vWAT and scWAT according to the relative metabolic rate and the metabolic demand 4 weeks after nutritional intervention [22]. Since we did not address the timing of FA mobility, but merely the end point values in the interventions, we cannot exclude the selective mobilization of specific FAs in response to the interventions at time-points preceding or exceeding those presented. Possibly in the same context, the observed lack of increase in serum FA during long-term fasting, is in line with previous observations on the transient nature of serum FA accumulation in response to fasting in the rats from our study [23] and others [24]. Other differences in outcomes between our study and those of others may result from the fact that in the present study rats were housed at thermoneutrality (28 °C) to render the outcomes more comparable to those of human studies [12,25]. Animals housed at thermoneutrality barely move, which has the advantage that the effect of mild exercise interventions can be individualized with more accuracy. Our data confirm those of a previous work in which total hepatic FA (not individually assessed) were significantly elevated after 48 h of fasting in rats housed in similar conditions (26 °C) [24]. Upon the previous analysis of whole-animal metabolic parameters and metabolic compounds in serum with respect to hepatic metabolism we observed that the increase in ketone production during fasting coincided with the reduced transcription of genes involved in hepatic lipogenesis [12], indicating that hepatic FAs must be supplied by external sources. We show here that the hepatic source of adipose FAs during fasting originates from subcutaneous, rather than visceral WAT. Interestingly, despite increased release of FA from vWAT in FE (including the highly abundant 16:0, 18:1, and 18:2), no increase in the measured FAs was observed in liver in FE compared to F. This can be explained by the fact that in our model the liver is transcriptionally programmed to boost FA oxidation and gluconeogenesis in FE [12]. Of note, hepatic levels of low-abundant FAs 14:0, 20:1 n-9, 20:2 n-6 did not correlate with their abundance and mobilization from WAT, indicating that these FAs are increasingly metabolized. The fasting-induced shift in hepatic proportion favoring the abundant FAs 18:1 n-9, 18:2 n-6 and 20:4 n-6 is explained by the fact that these FAs accumulate to a greater extent with respect to 16:0 and 18:0. Given that all mentioned FA individually accumulate, and their mobilization from scWAT and vWAT is similar, this may imply that 16:0 and 18:0 are preferentially used as substrates with respect to 18:1 n-9, 18:2 n-6 and 20:4 n-6. These data are in line with those found in 16 h fasted mice [26]. The observed increase in visceral adipose fuel channeling to the liver in response to FE provides an additional energy source to surrounding organs. This may, at least in part, explain: (1) the beneficial effects observed in the muscle [27], brain [27], and gonads [28] in the same model, and (2) the amelioration of body composition in response to a similar intervention were carried out in healthy males [29].

The observed differential response of ATGL and HSL in the separate adipose tissues to the interventions carried out in this study is striking. A previous paper brought to light a delicate interplay between ATGL and HSL in inducing FA release and that this depends on AMP-activated protein kinase (AMPK) [30]. In a human white adipocyte model, ATGL, not HSL, was identified as being responsible for FA release from the adipocyte, despite the capacity of both lipases to produce free FA through triglyceride (ATGL) or diglyceride (HSL)-specific hydrolase activity [31]. Indeed, it has recently been demonstrated that ATGL is indispensable for hepatic ketone production by providing PPARα ligands during fasting [32]. In line with these key observations, it may be hypothesized that only in cases in which ATGL protein exceeds HSL Ser^563^, FA mobilization during fasting occurs. We show here that this condition exists in fasted scWAT, but not vWAT, in which HSL and ATGL are both active. If the above hypothesis is correct, the exercise-induced FA mobility in vWAT during fasting may be the result of the observed decreased HSL Ser^563^ and increased Ser^565^ phosphorylation, inactivating the enzyme. In this context, a previous study showed that in fasted rats 57% of lipolyzed free fatty acid (FFA) are re-esterified back into adipose triglycerides (TG), the majority of which are produced from lipase-produced FAs before their release in the plasma [33]. This process may be primarily governed by vWAT since it has been claimed that human scWAT in the fasting state does not take up FA [34]. Proteomic analysis in mice has revealed that intermittent fasting dramatically and selectively reduces the lipolysis pathway in vWAT, despite the increased phosphorylation of HSL at Ser^660^ during 16 h of fasting [35], the latter result being in line with our data. Although data on human visceral adipose tissue are lacking, it has been shown that the response to fasting in human subcutaneous adipose tissue does not involve HSL Ser^563^ phosphorylation, with ATGL protein being increased [36], which is in line with the results of the current study and would explain the lack of FA re-uptake in scWAT [34] should the above hypothesis prove to be correct. We observed that induction of ATGL protein in scWAT, though correlating with a tendency of AMPK protein levels to increase, is not associated with its phosphorylation at Thr^172^. A Lack of AMPK phosphorylation during starvation in scWAT has also been observed in mice [37]. In contrast, we found that the ATGL protein in vWAT in both F and FE is associated with AMPK phosphorylation at Thr^172^, confirming the correlation between starvation-induced AMPK phosphorylation [37] and 5-Aminoimidazole-4-carboxamide ribonucleotide (AICAR)-induced AMPK phosphorylation and ATGL protein levels in visceral adipocytes [30]. Interestingly, it has been shown that the prolonged AICAR treatment of visceral adipocytes reduced the phosphorylation of HSL at Ser^563^ and increased HSL Ser^565^ phosphorylation [30], which is reminiscent of the effect observed in FE. Since the same study revealed that in vivo treatment with AICAR for 8 h in male Wistar rats induced serum FA accumulation [27], this provides additional clues regarding the fact that increased FA mobility in FE may involve increased ATGL activity over that of HSL. More extensive studies need to be performed to elucidate this further.

We have shown in a previous work on the same model that the reduction in vWAT mass in F has been shown to be similar to that observed in FE [12]. Thus, a lack of net FA mobilization in vWAT in the F condition does not imply unaltered tissue mass since FA are rapidly oxidized [27], re-esterified into triglycerides [30], and simultaneously produced ex-novo through hydrolysis of triglycerides and diglycerides by ATGL and HSL. Importantly, the FE-related release of FA from vWAT, preventing energy storage in the adipocyte, may result in a sustained reduction in visceral fat mass, and may prevent the so-called “jojo effect”. This is of importance considering that the surgical removal of VAT in rats induced the effect of caloric restriction on longevity by about 20% by enhancing insulin signaling [38]. Of note, one limitation of this study concerns the fact that we analyzed FA, without discriminating between free FA and those associated in TAGs. Therefore, we cannot pinpoint different functions for these two forms of fatty acids that remain in the tissue. However, the decrease of the FA content in the tissues is due to release of free FA in response to lipase activity, which at any rate allows to address their mobility. To our knowledge, this is the first study to provide detailed FA analysis in different tissues during the metabolic switch induced by either fasting, or exercise or both and to correlate adipose depot-specific FA release with differential interplay between ATGL and HSL in a rodent model. The obtained results provide insight on how subtle physiological interventions can change visceral adipose modeling, with consequences for the entire organism. We have previously studied the effect of a similar intervention in male subjects and found that body composition improved [29]. Based on the results of our present study, future studies in human subjects should address the question whether the effect on visceral fat mass-loss with combined exercise and fasting will be more prolonged with respect to fasting or exercise only. Results may lead to possible clinical applications.

## Data Availability

The data presented in this study are available on request from the corresponding author.

## Supplementary Materials

The following supporting information can be downloaded at: https://www.mdpi.com/2072-6643/15/14/3095/s1, Figure S1: Proportions of each measured FA in scWAT, Figure S2: Proportions of each measured FA in vWAT, Figure S3: Proportions of each measured FA in liver, Figure S4: Proportions of each measured FA in serum.

## Abbreviations

C: chow-fed: sedentary controls, E: exercised, F: fasted, FE: fasted and exercised. SFA, saturated fatty acids; MUFA, mono unsaturated fatty acids; n6PUFA, n6 poly unsaturated fatty acids; n3 PUFA, n3 poly unsaturated fatty acids; TFA, trans fatty acids; vWAT: visceral white adipose tissue; scWAT: subcutaneous white adipose tissue.
