# Supplementary material for: Exercise Equals the Mobilization of Visceral versus Subcutaneous Adipose Fatty Acid Molecules in Fasted Rats Associated with the Modulation of the AMPK/ATGL/HSL Axis
Source: Nutrients. 2023 Jul 10;15(14):3095. doi: 10.3390/nu15143095 (PMC10386727; doi:10.3390/nu15143095)
Supplement: Supplementary file 1 [file nutrients-15-03095-s001.zip › Figure S3.pdf]

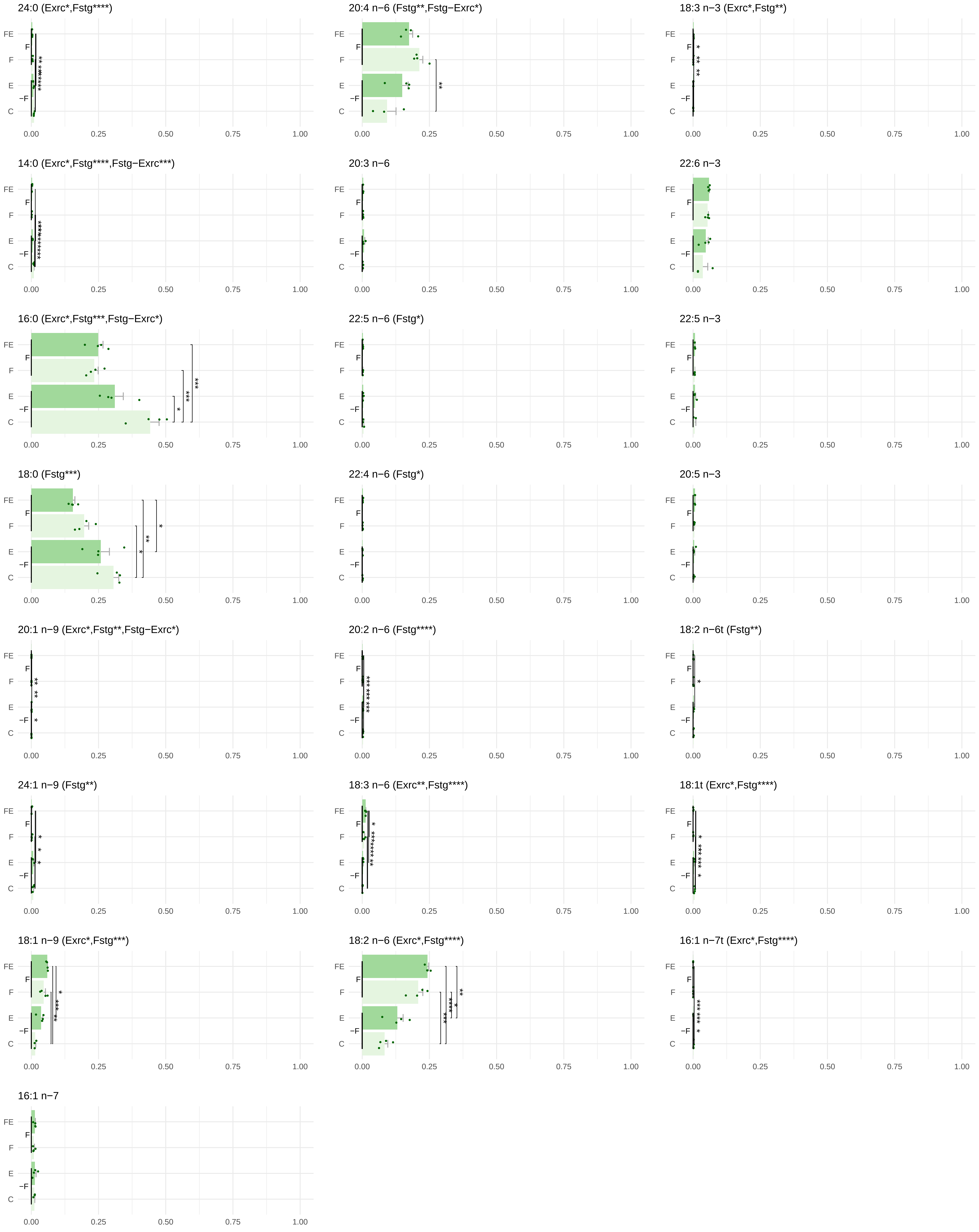
24:0 (Exrc*,Fstg****)
20:4 n−6 (Fstg**,Fstg−Exrc*)
18:3 n−3 (Exrc*,Fstg**)
14:0 (Exrc*,Fstg****,Fstg−Exrc***)
20:3 n−6
22:6 n−3
16:0 (Exrc*,Fstg***,Fstg−Exrc*)
22:5 n−6 (Fstg*)
22:5 n−3
18:0 (Fstg***)
22:4 n−6 (Fstg*)
20:5 n−3
20:1 n−9 (Exrc*,Fstg**,Fstg−Exrc*)
20:2 n−6 (Fstg****)
18:2 n−6t (Fstg**)
24:1 n−9 (Fstg**)
18:3 n−6 (Exrc**,Fstg****)
18:1t (Exrc*,Fstg****)
18:1 n−9 (Exrc*,Fstg***)
18:2 n−6 (Exrc*,Fstg****)
16:1 n−7t (Exrc*,Fstg****)
16:1 n−7
FE
F
E
C
0.00
0.25
0.50
0.75
1.00
