# Supplementary figures and images for: Exercise Equals the Mobilization of Visceral versus Subcutaneous Adipose Fatty Acid Molecules in Fasted Rats Associated with the Modulation of the AMPK/ATGL/HSL Axis
Source: Nutrients. 2023 Jul 10;15(14):3095. doi: 10.3390/nu15143095 (PMC10386727; doi:10.3390/nu15143095)

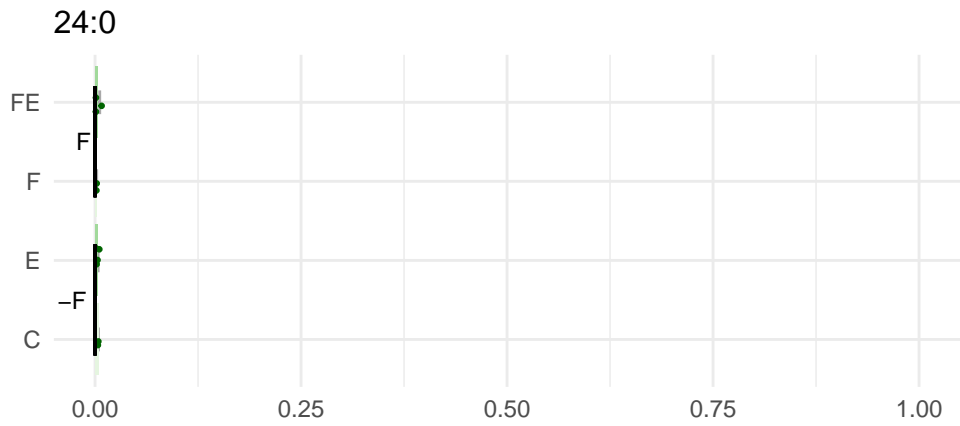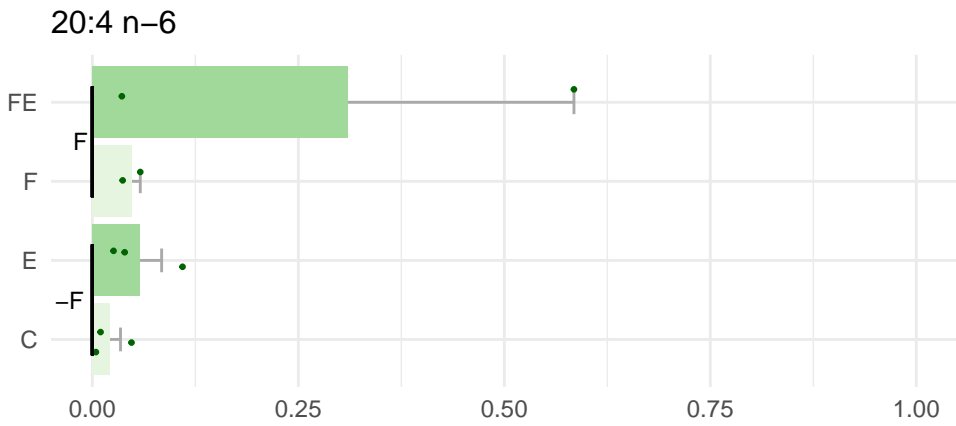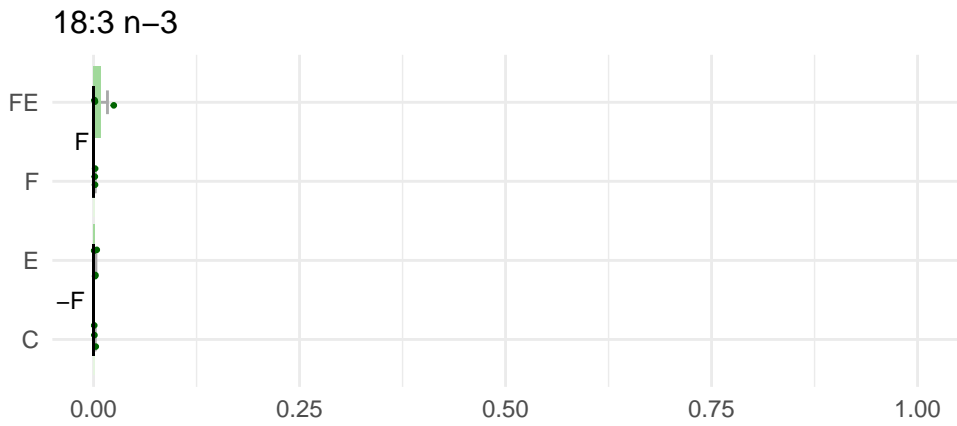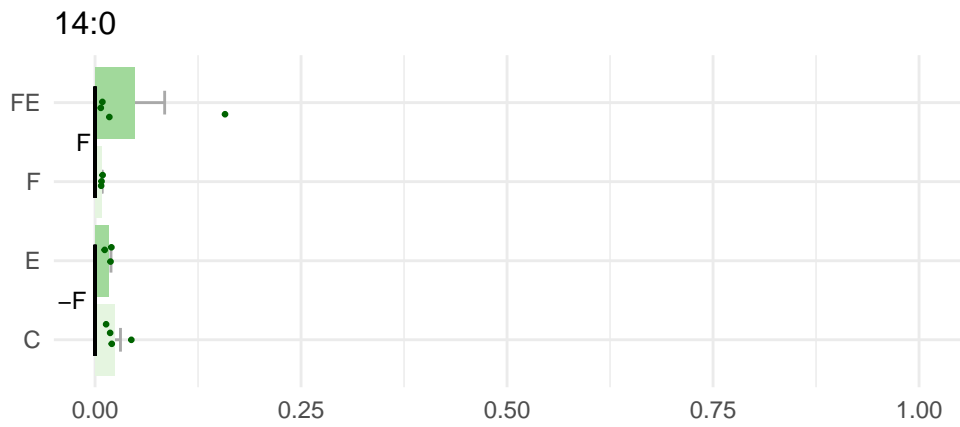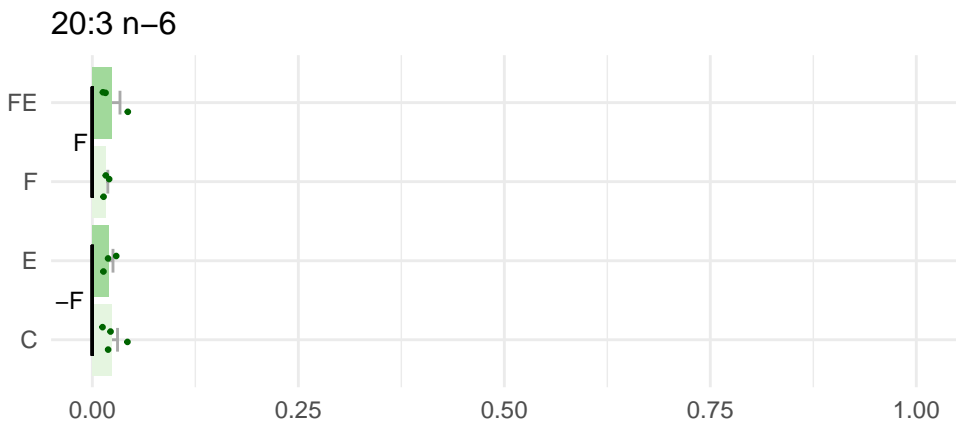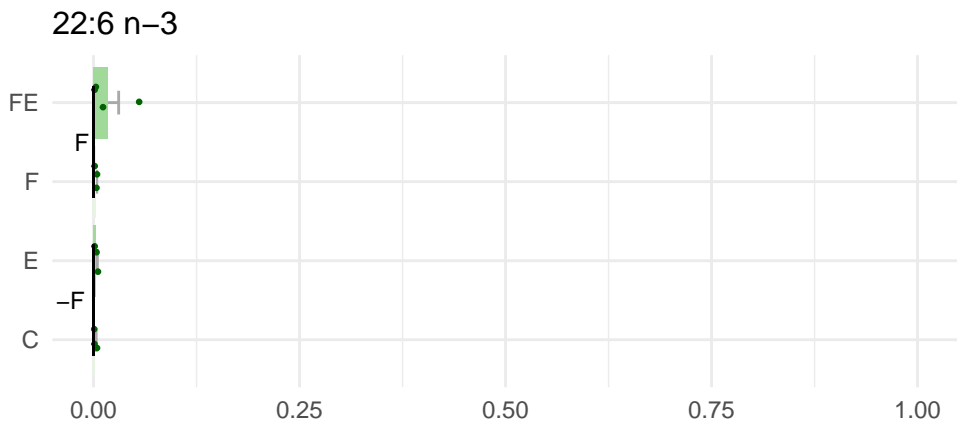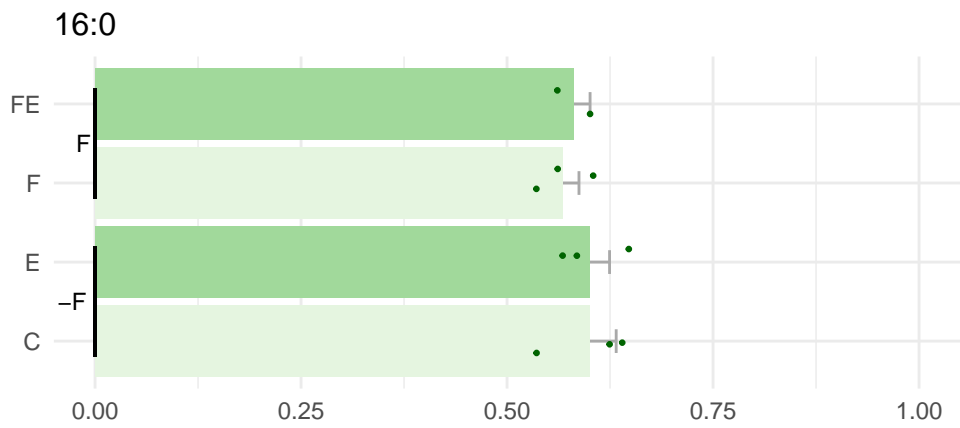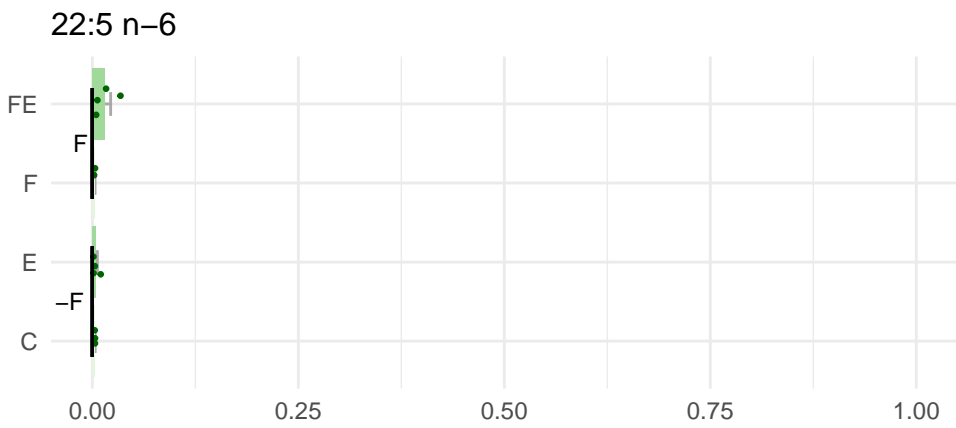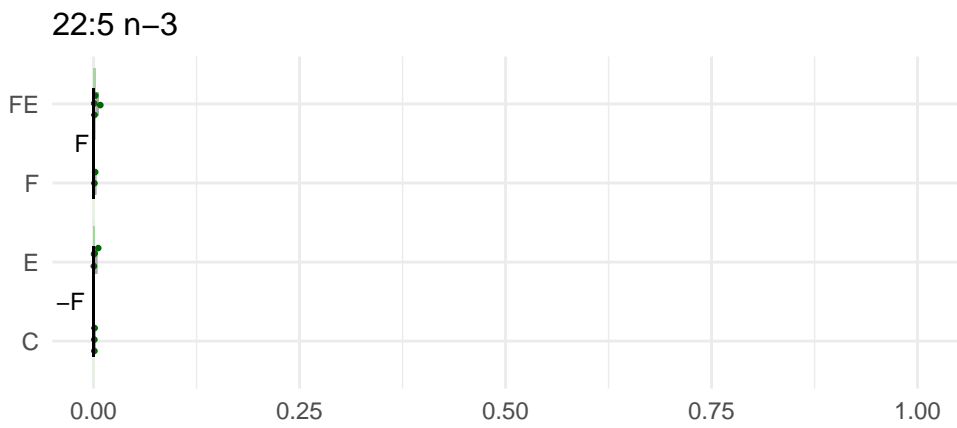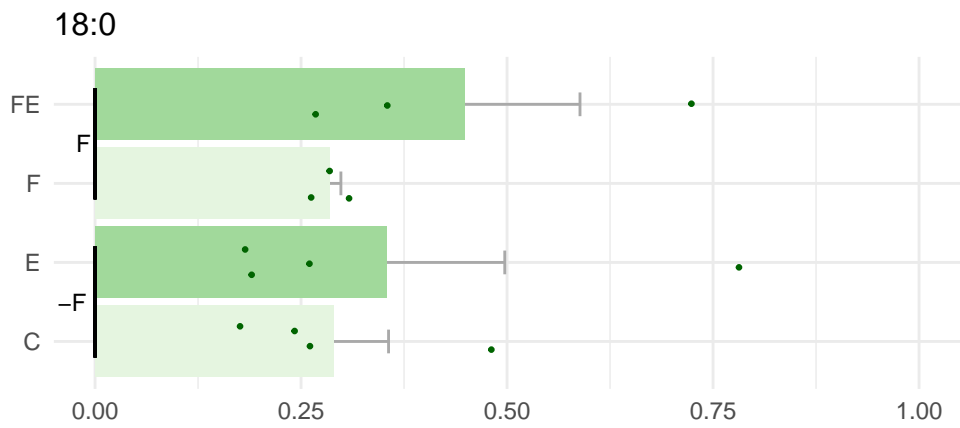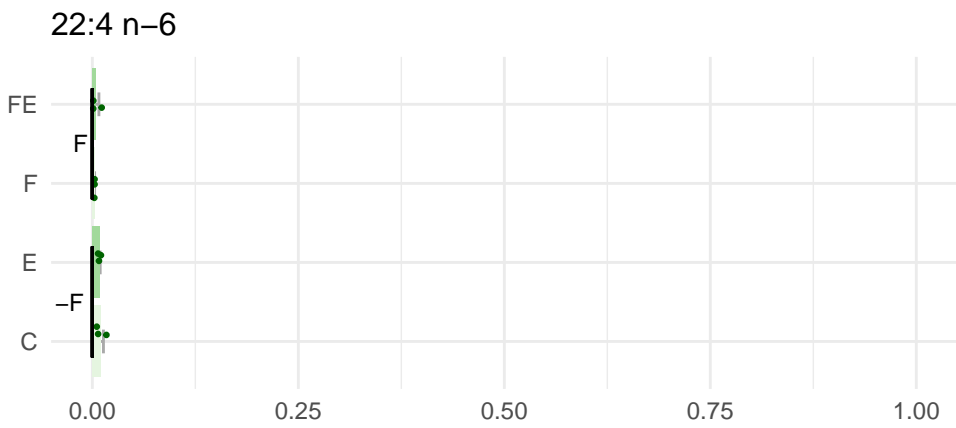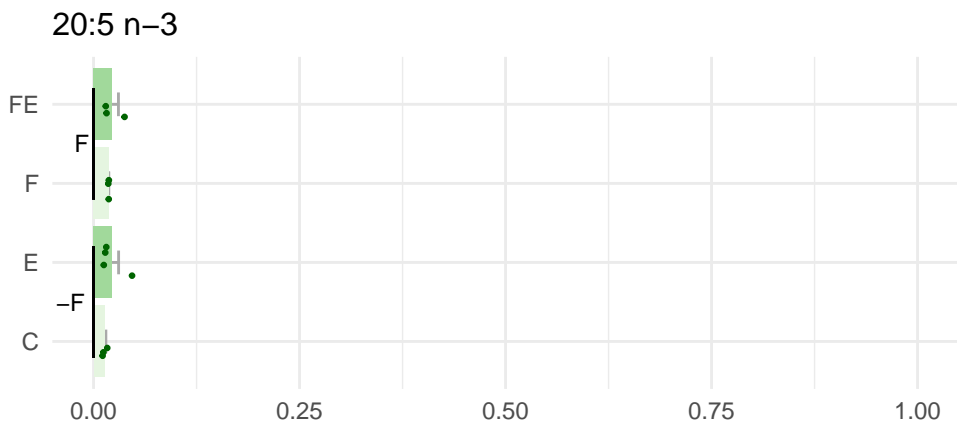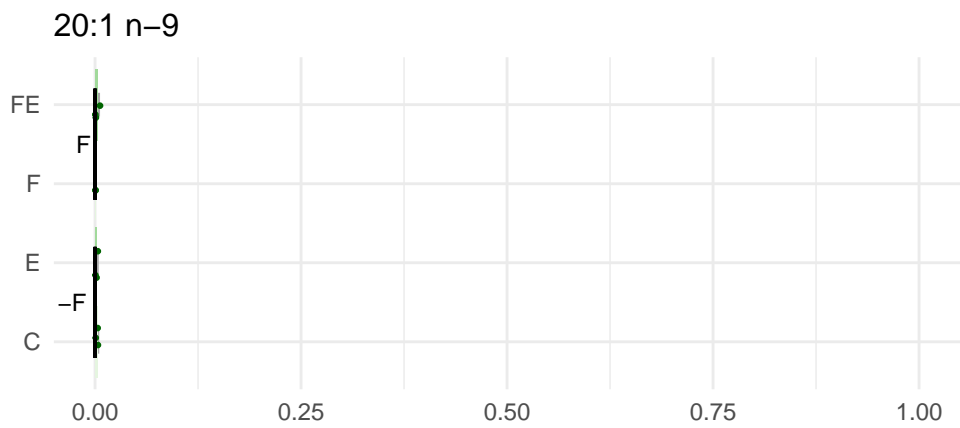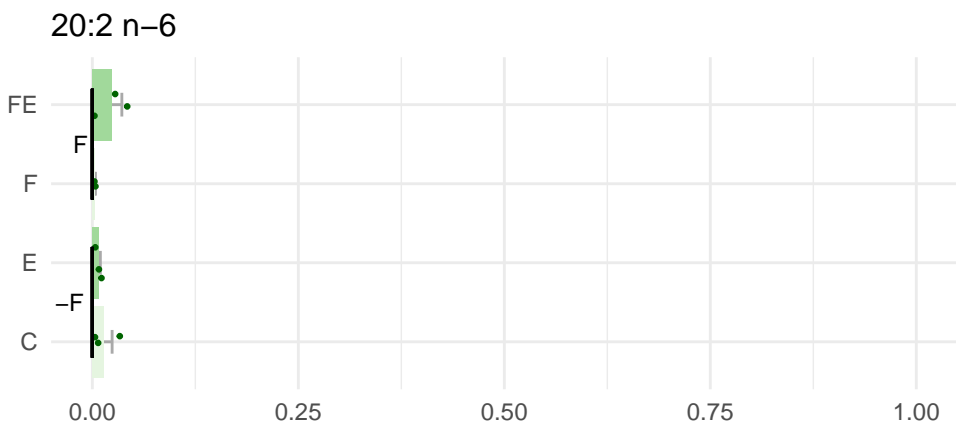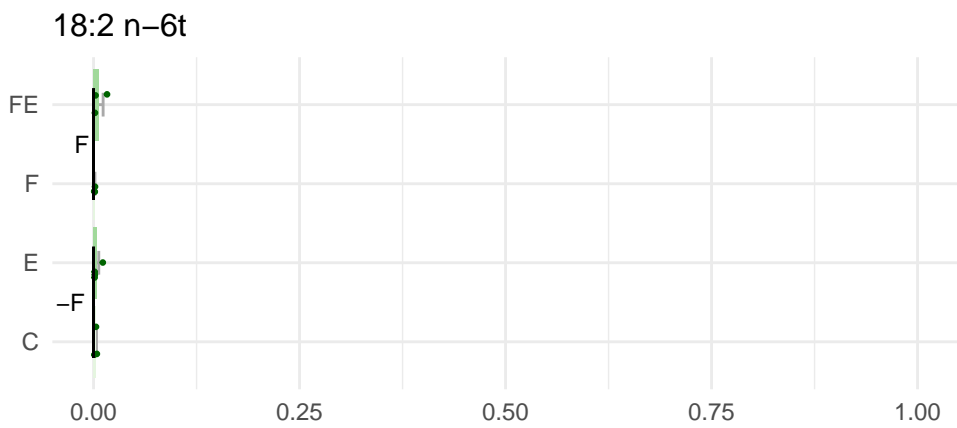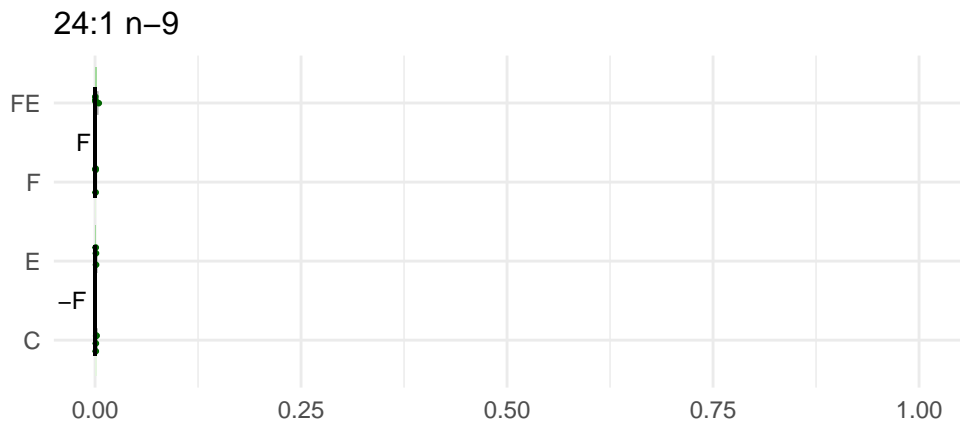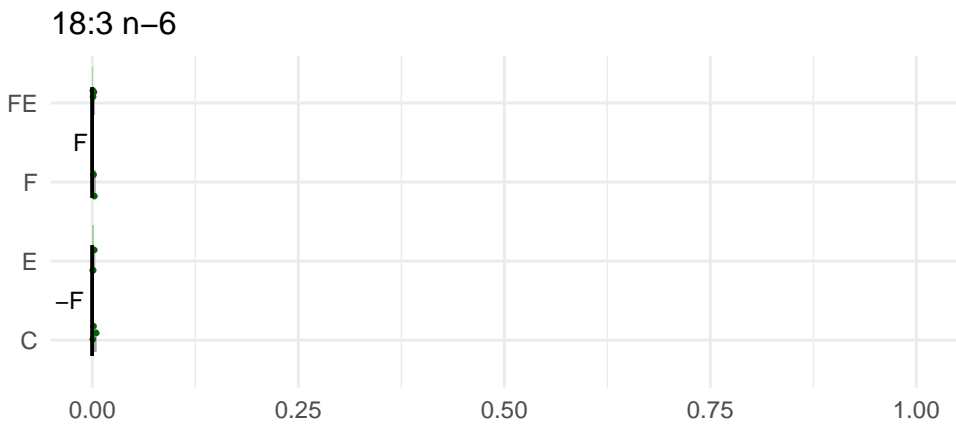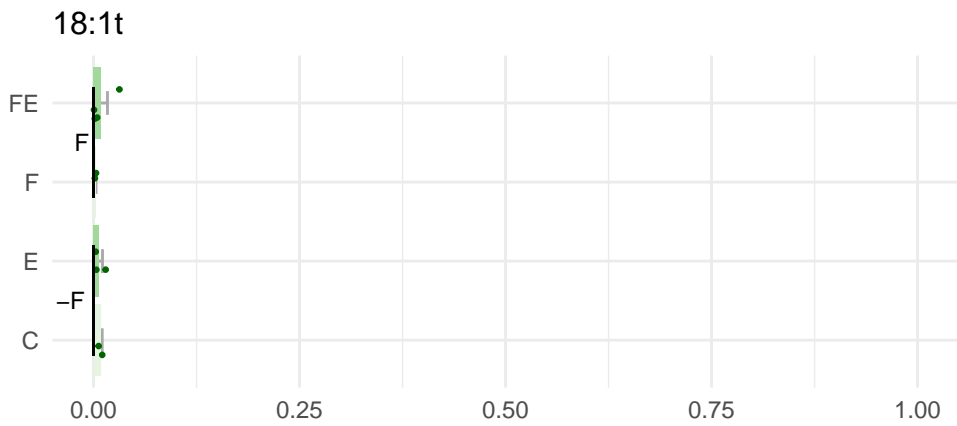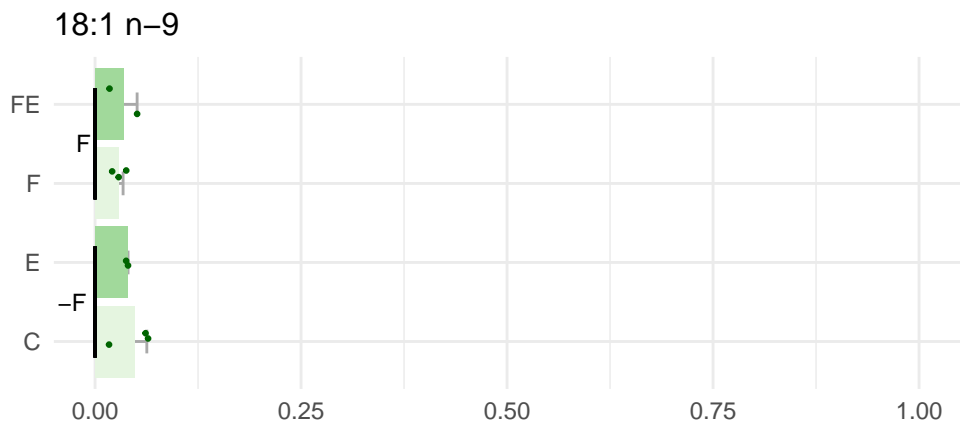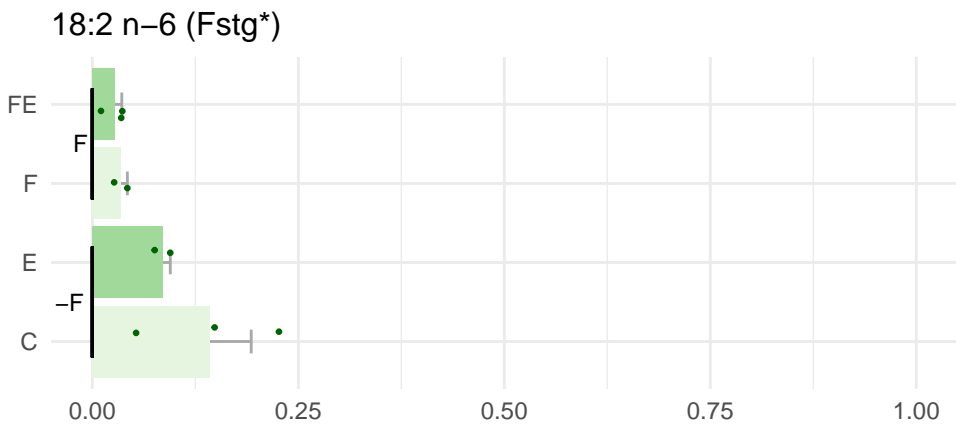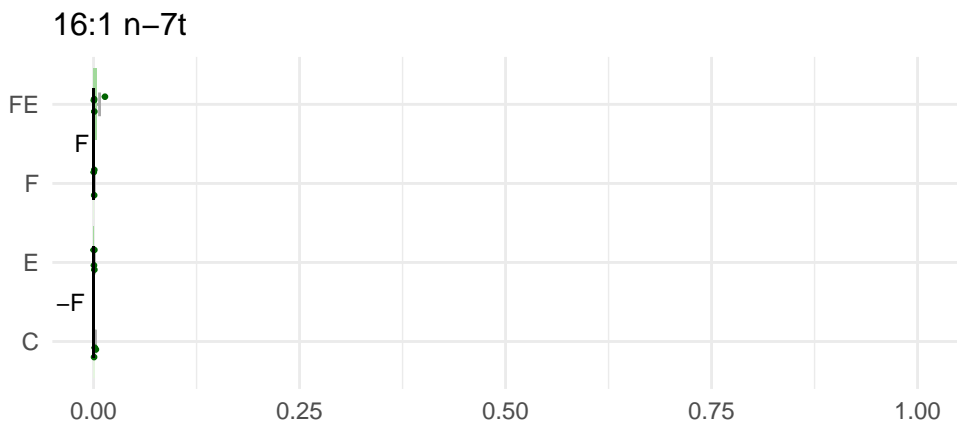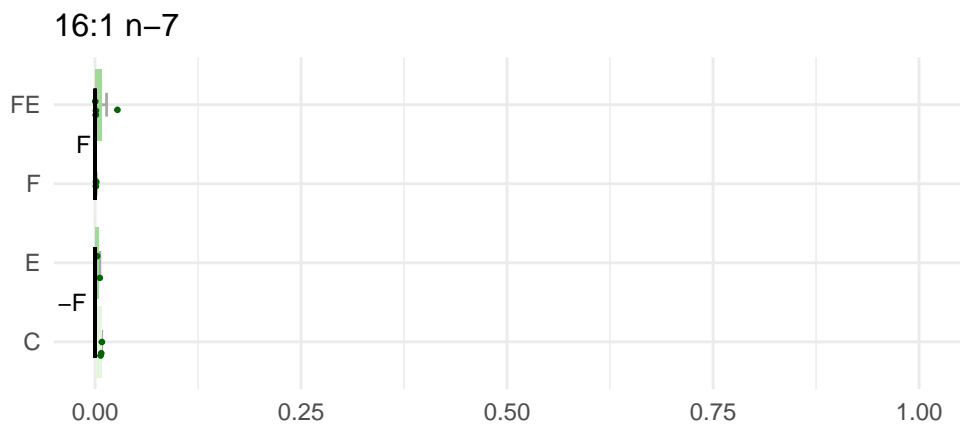

Supplement: Supplementary file 1 [file nutrients-15-03095-s001.zip › Figure S4.pdf]

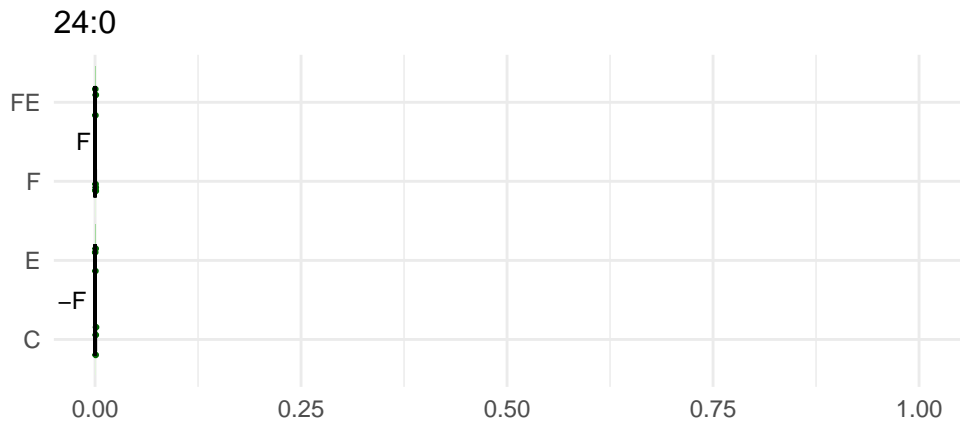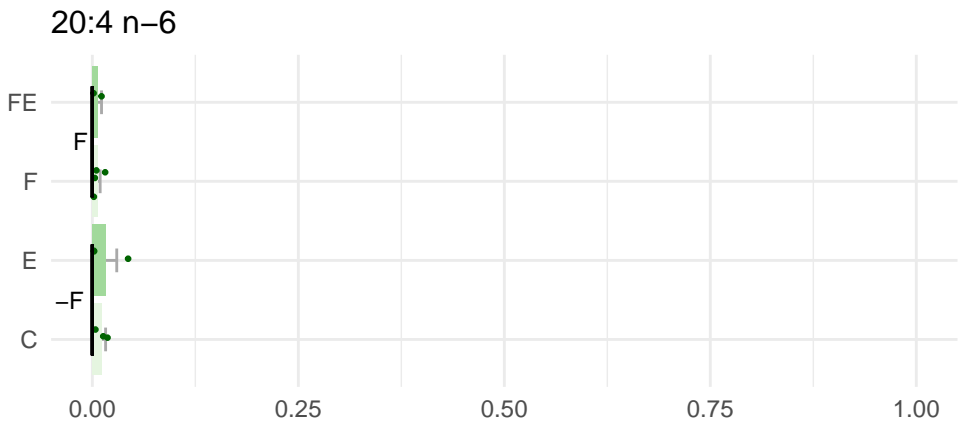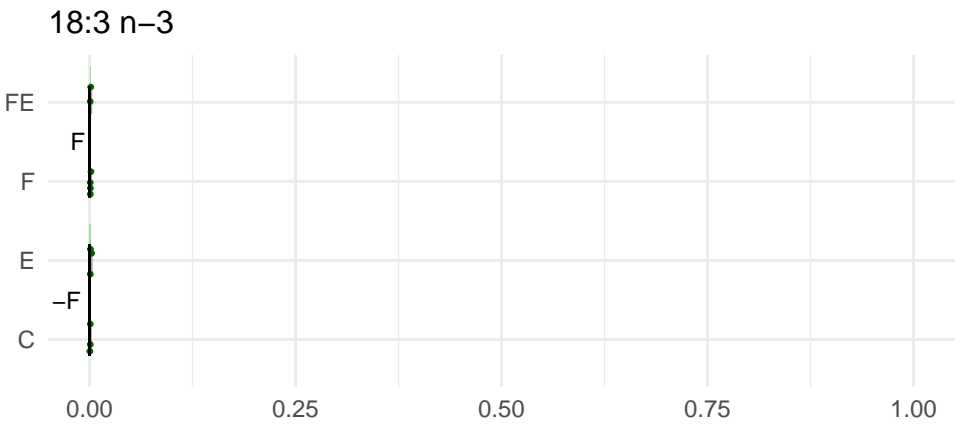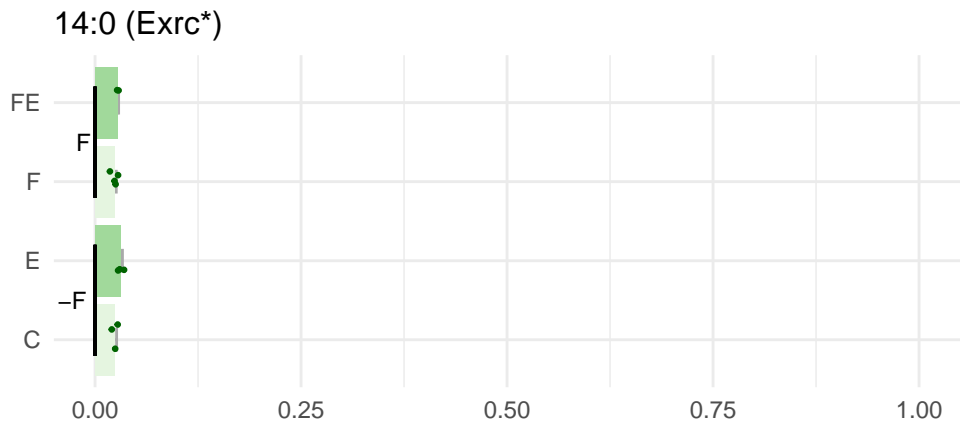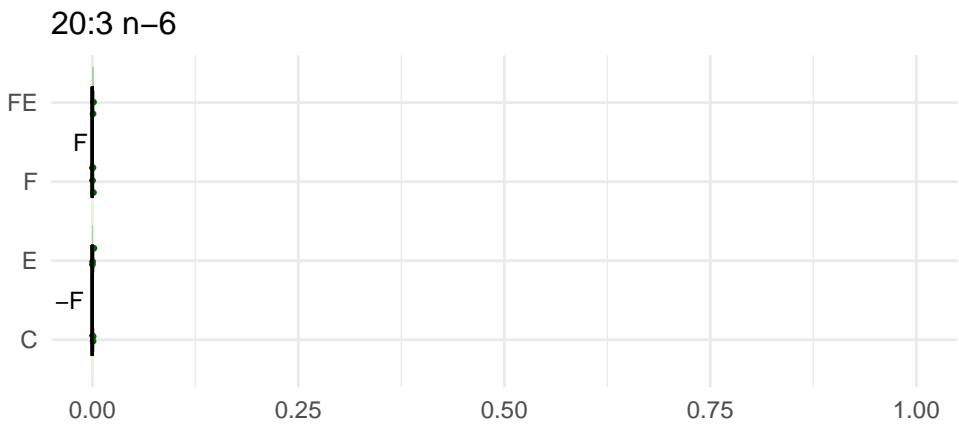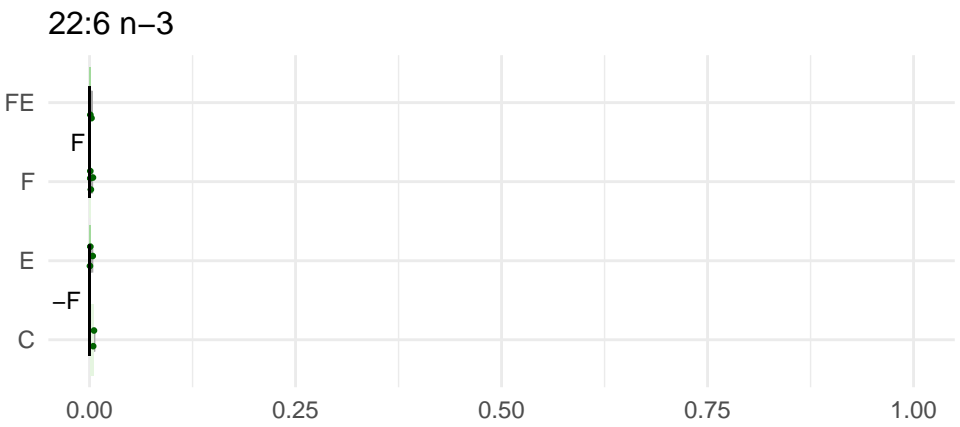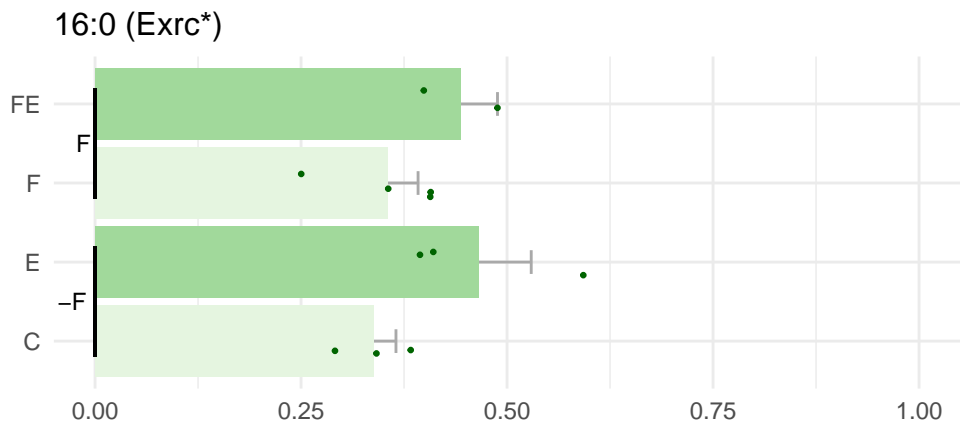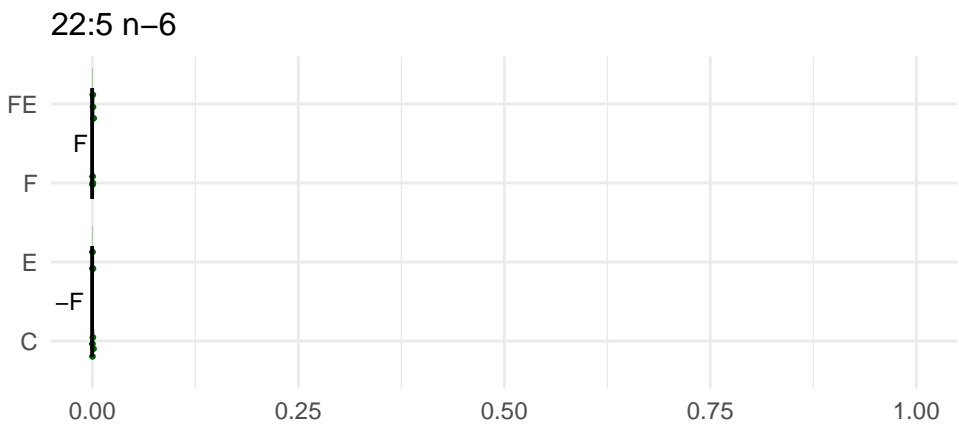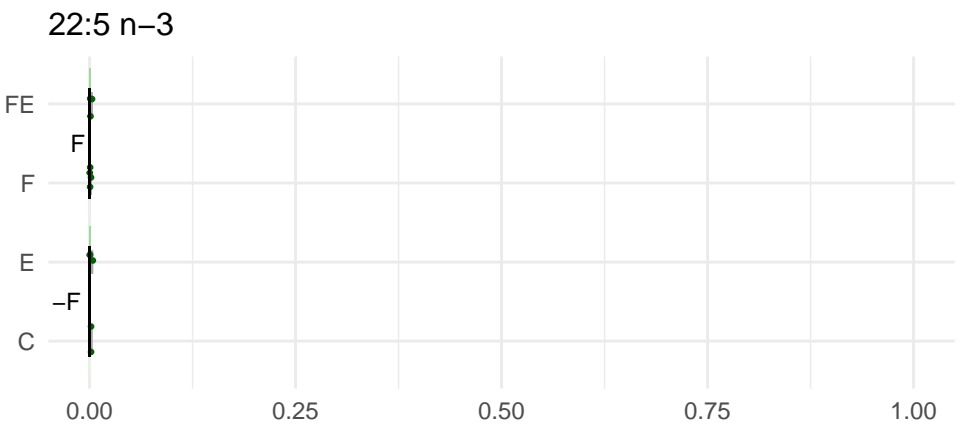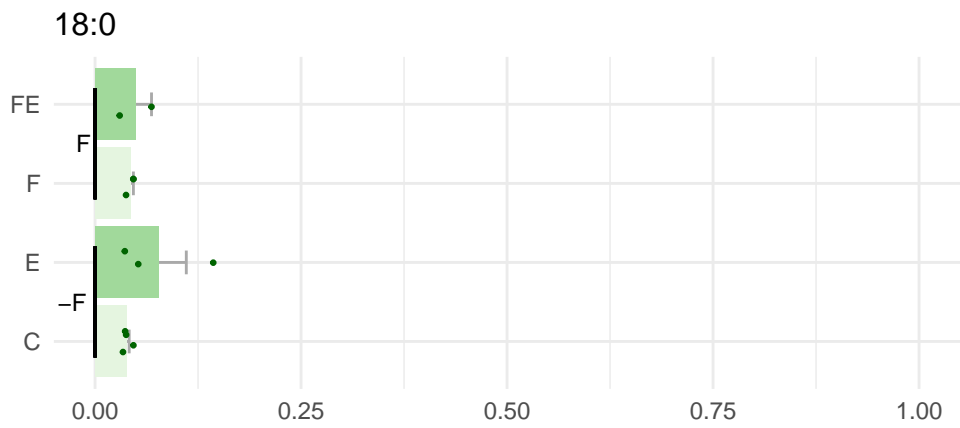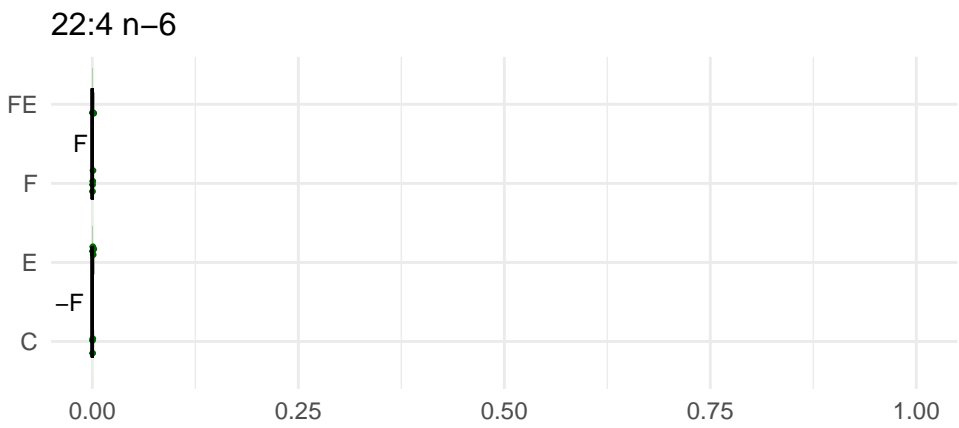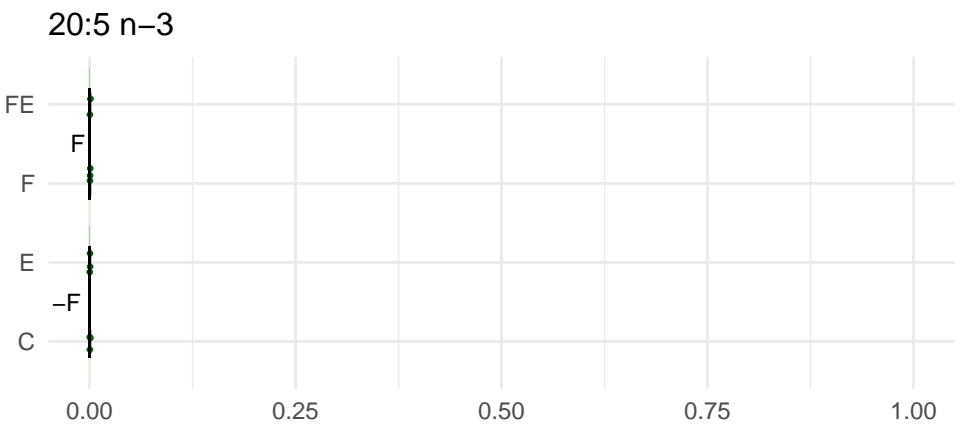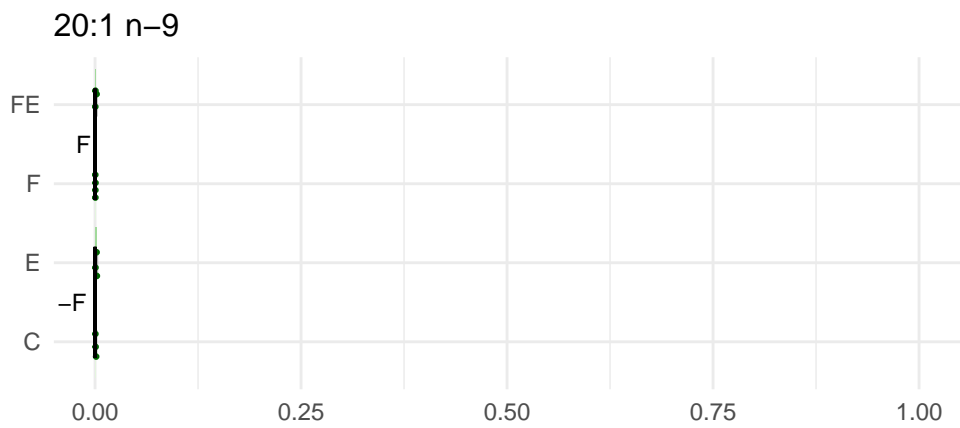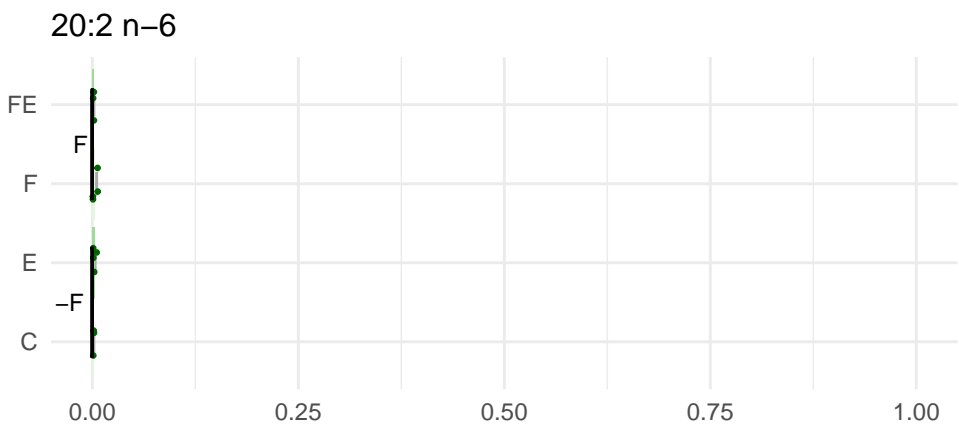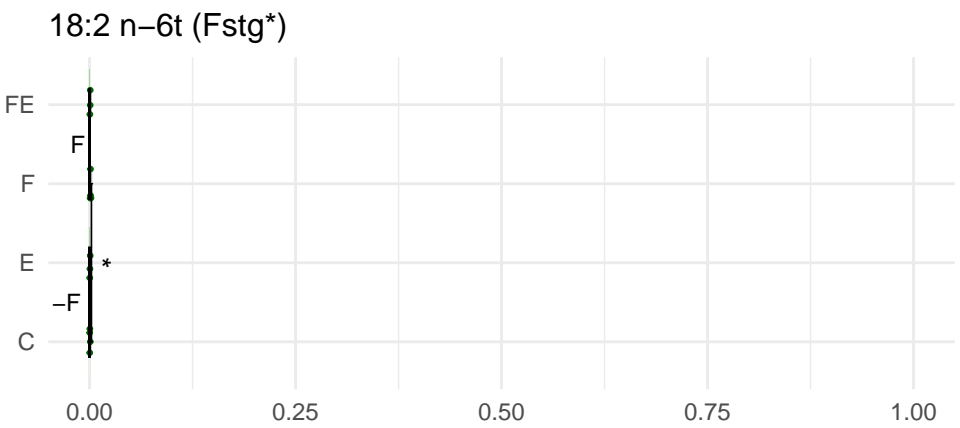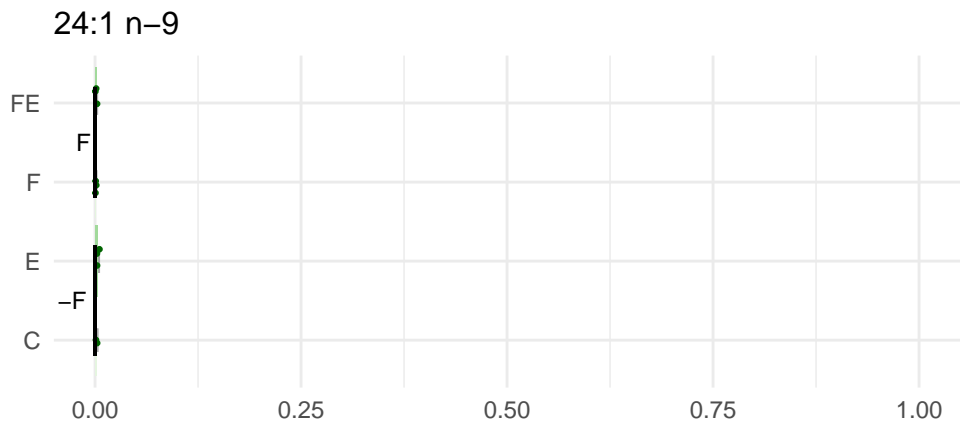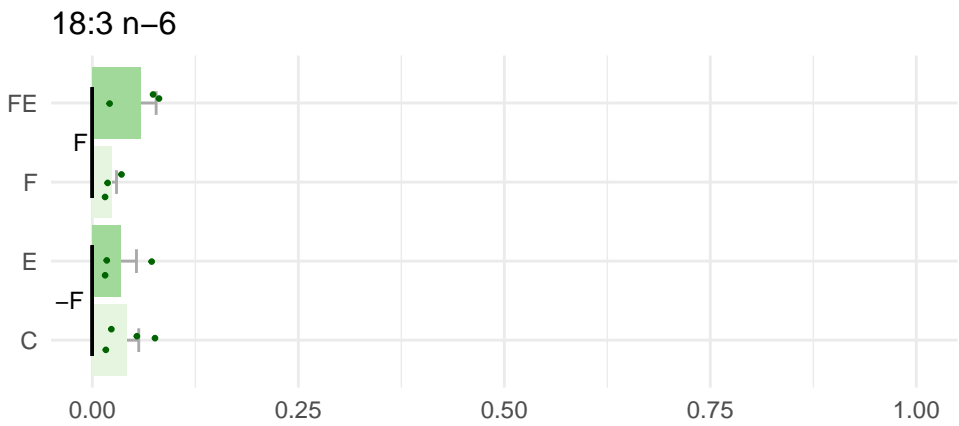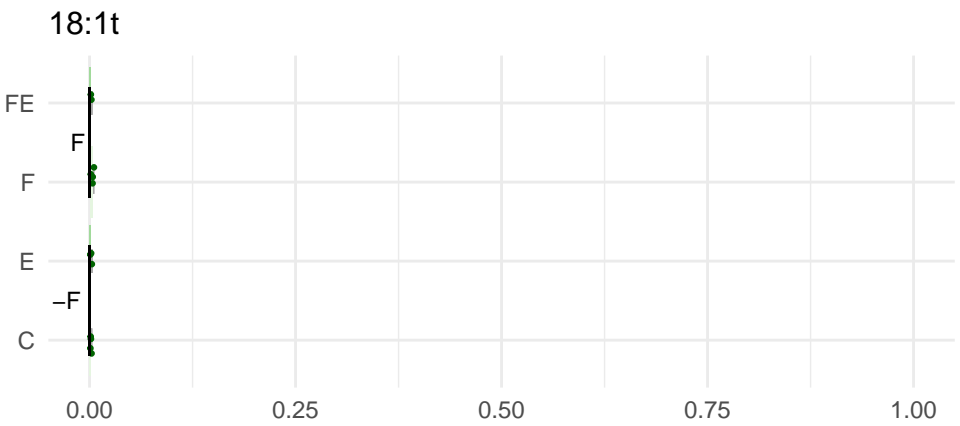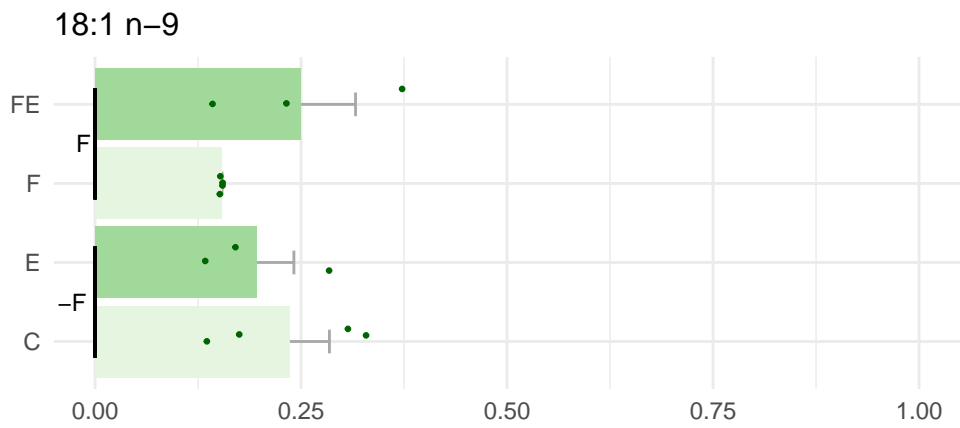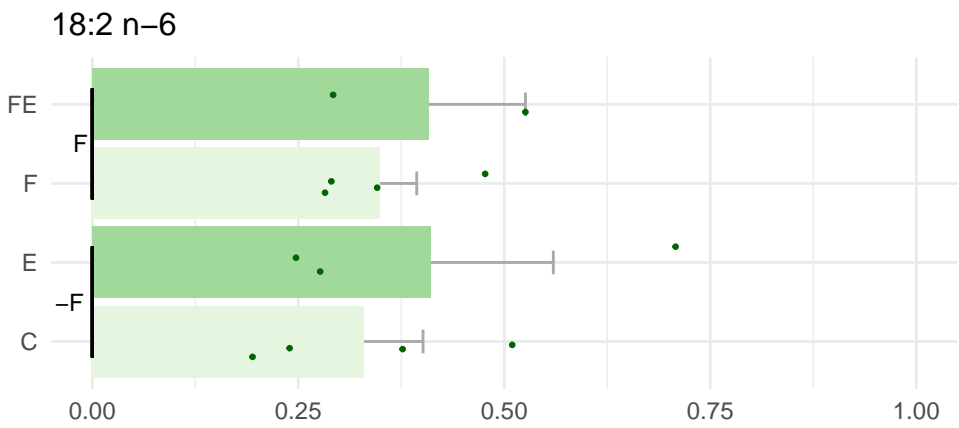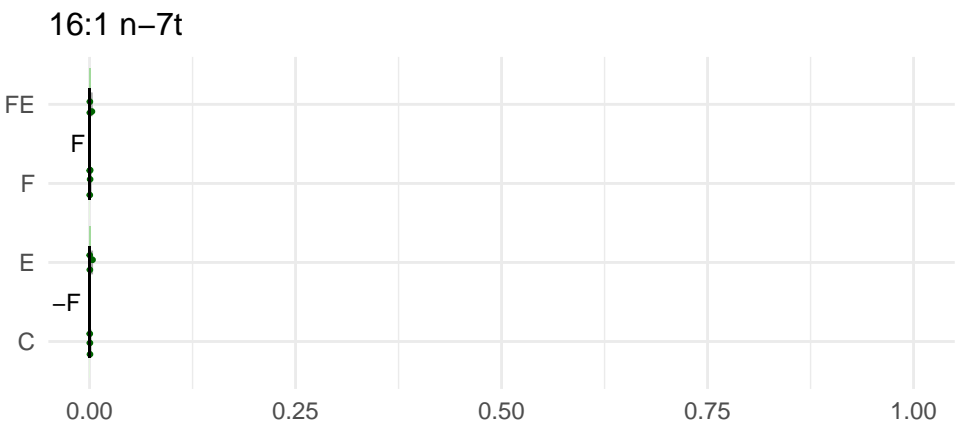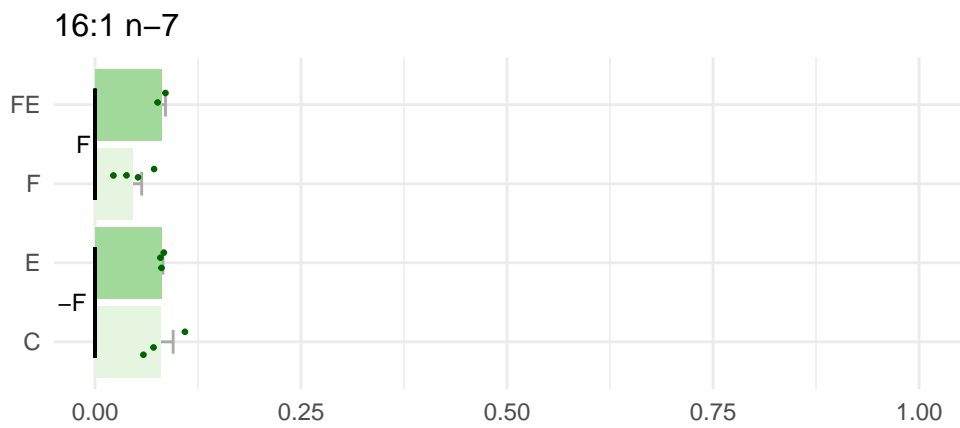

Supplement: Supplementary file 1 [file nutrients-15-03095-s001.zip › Figure S1.pdf]

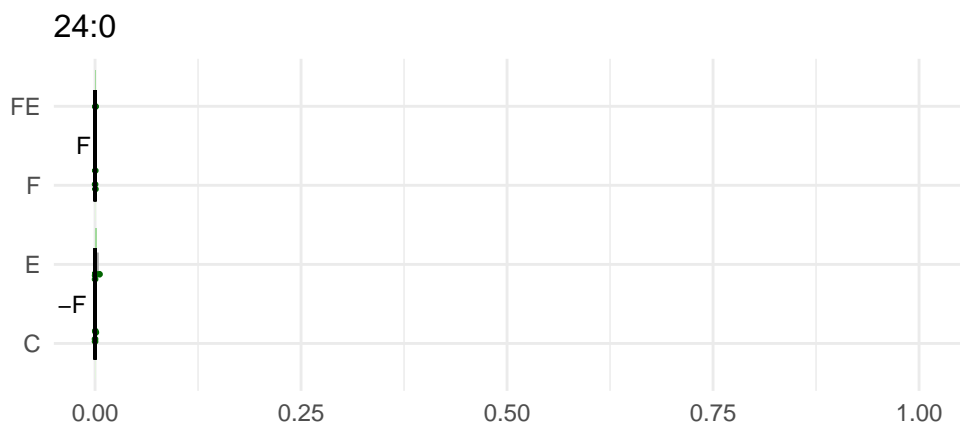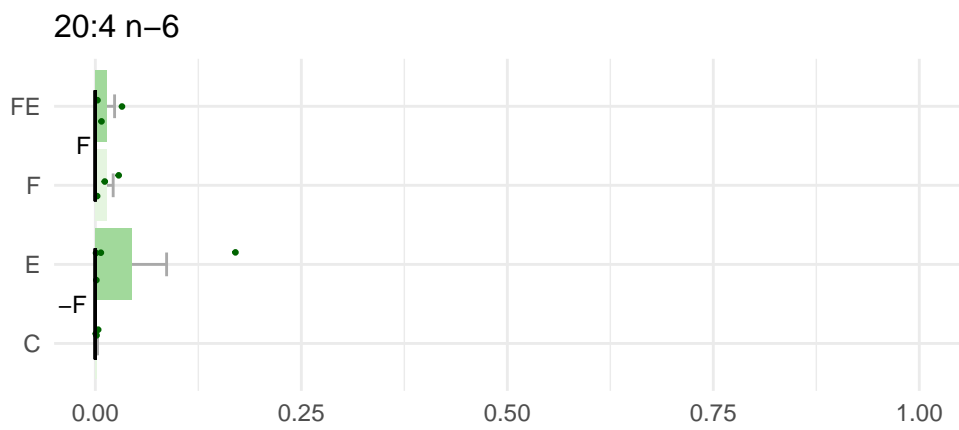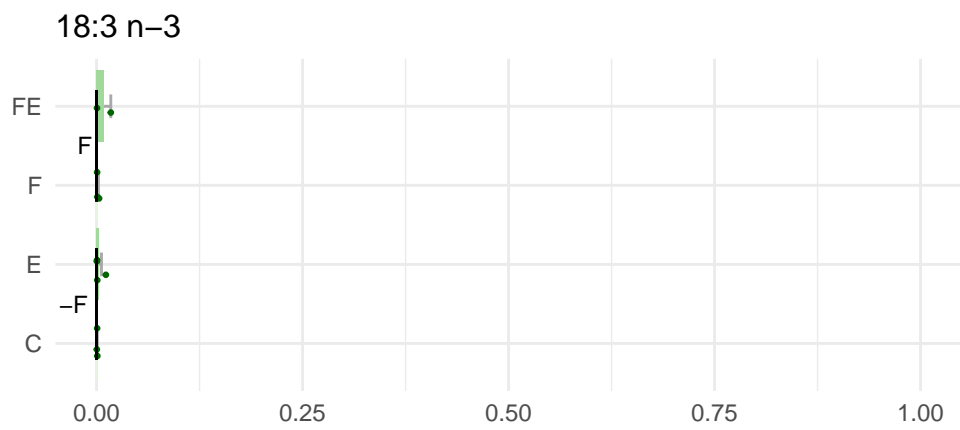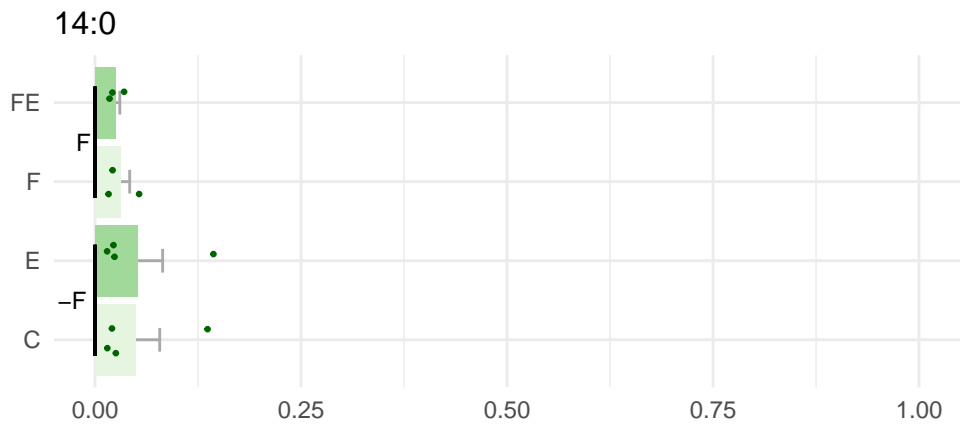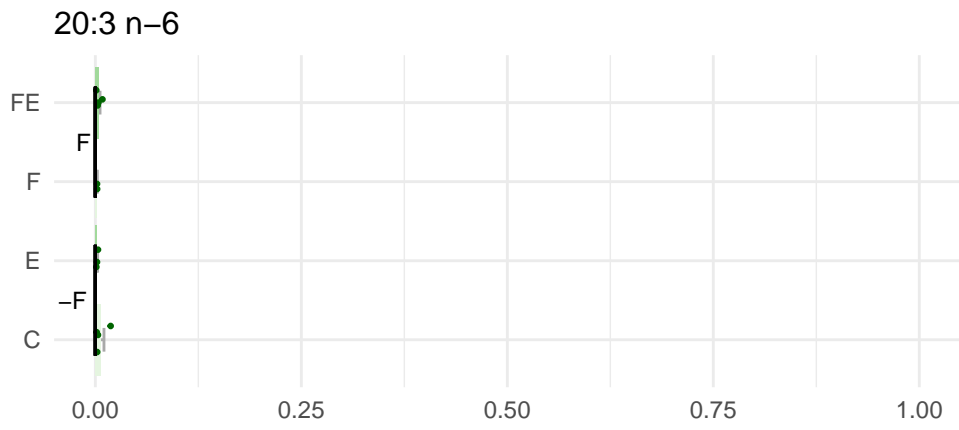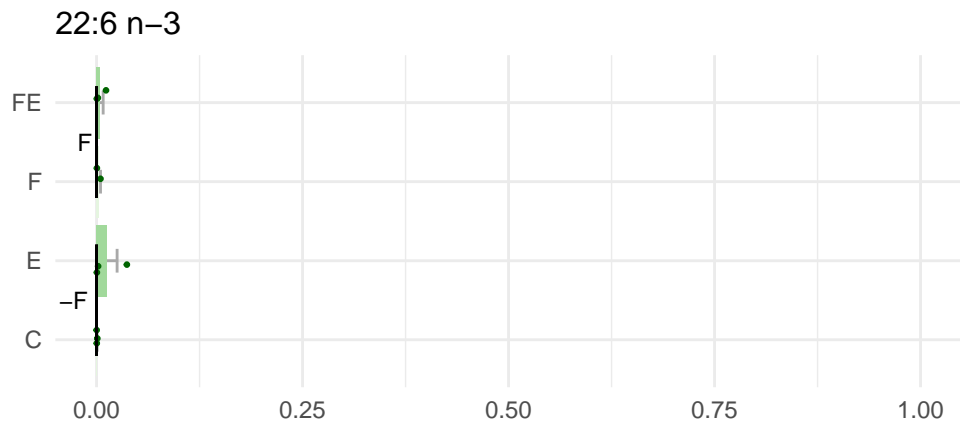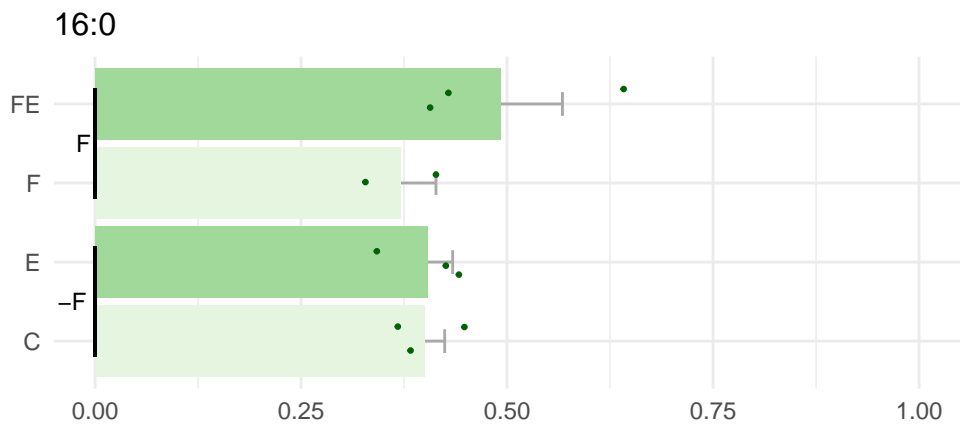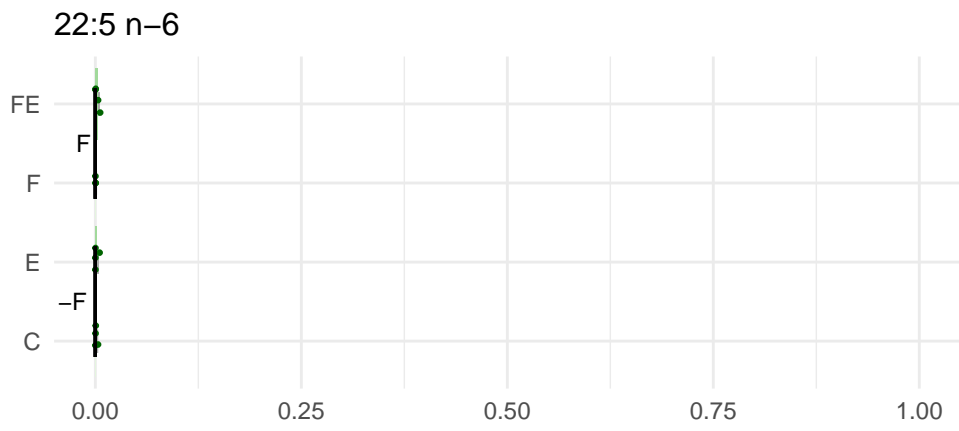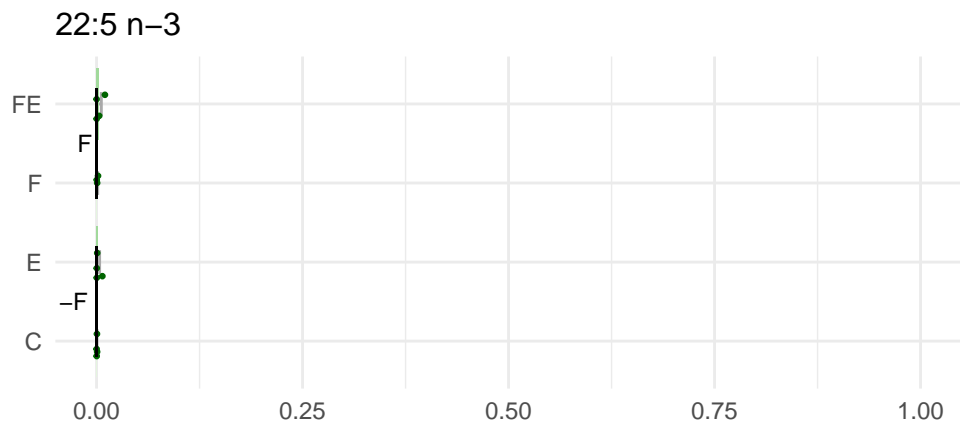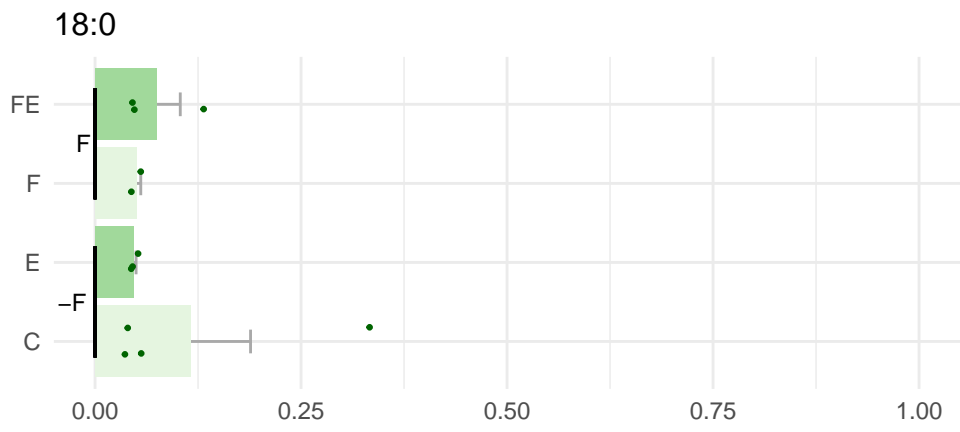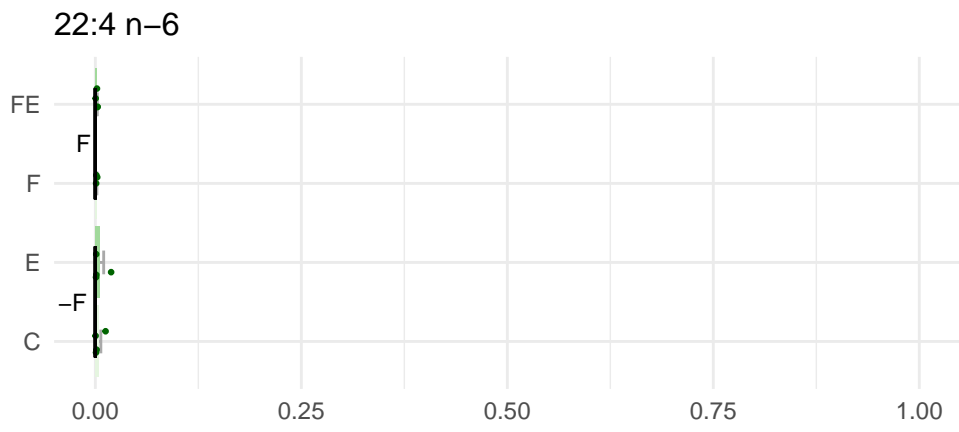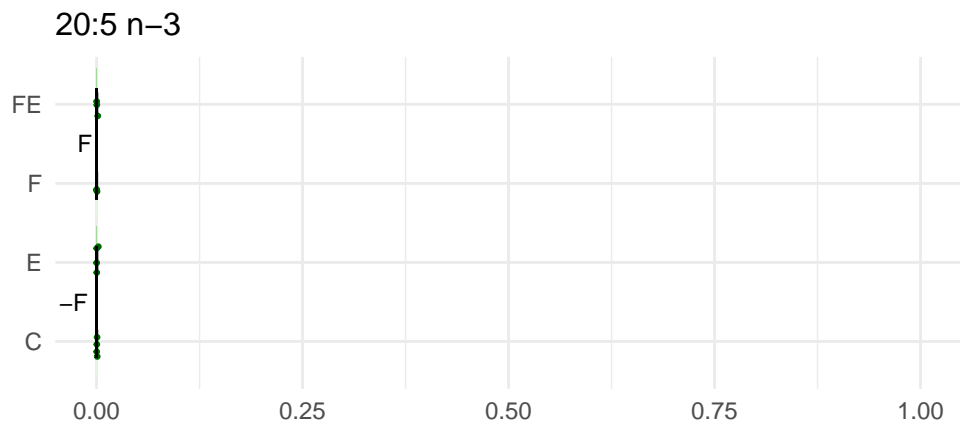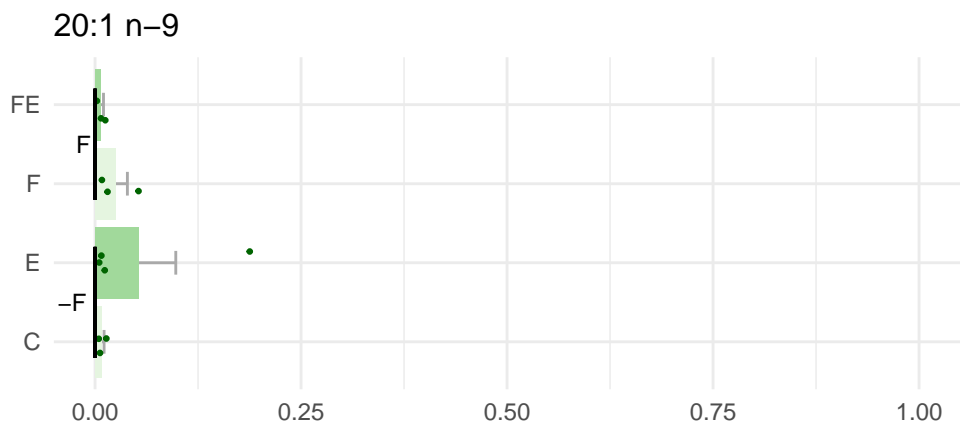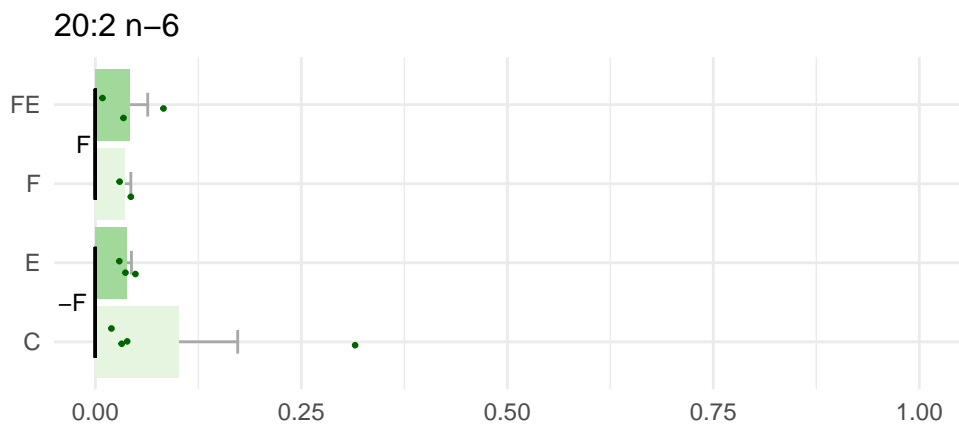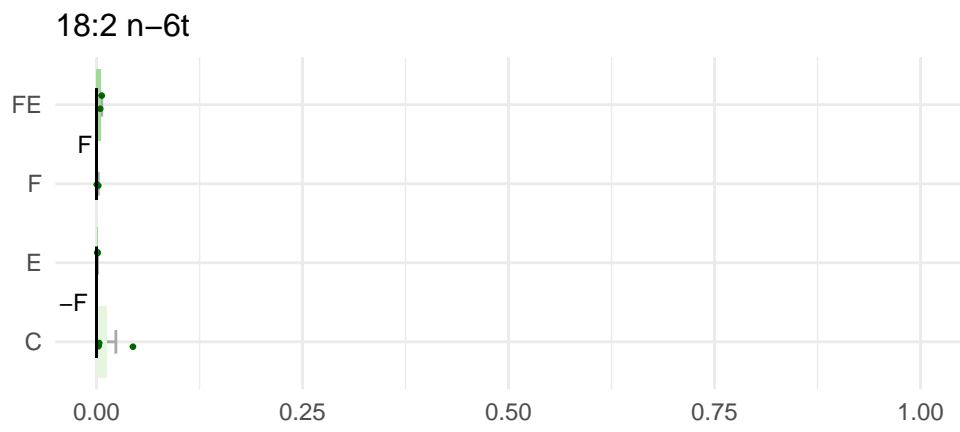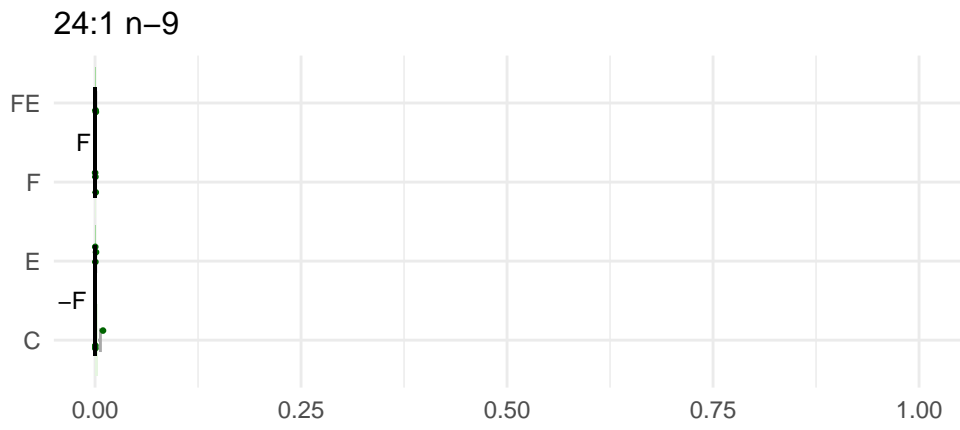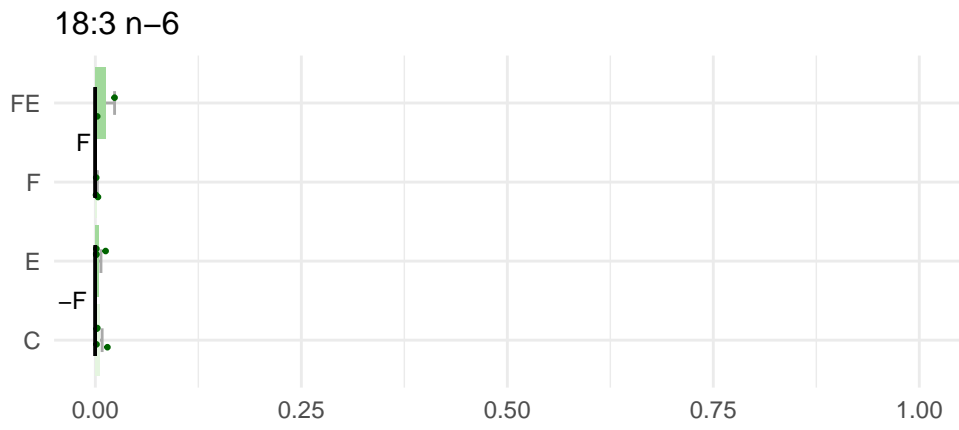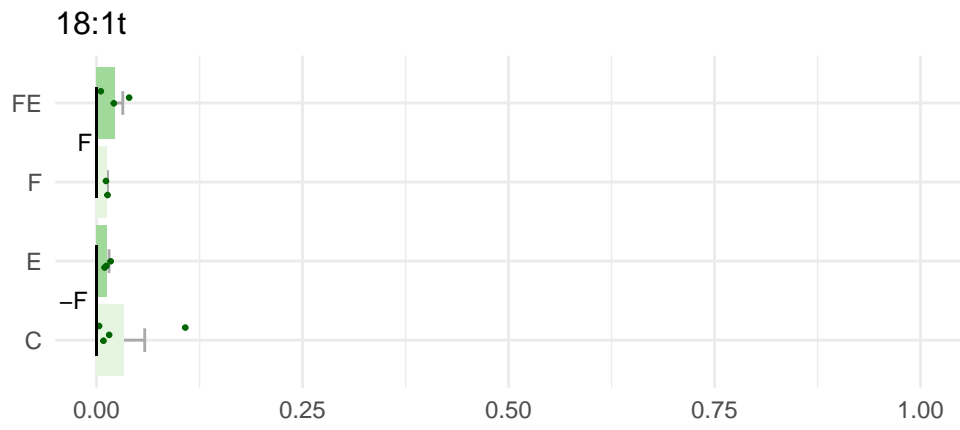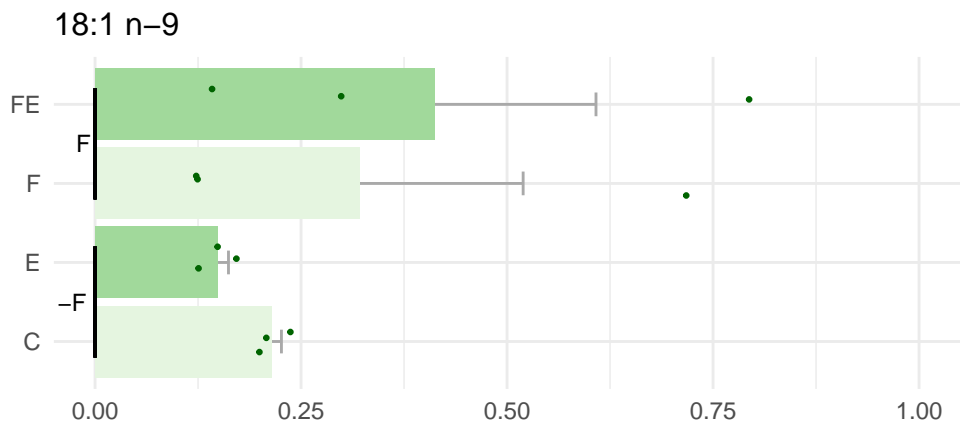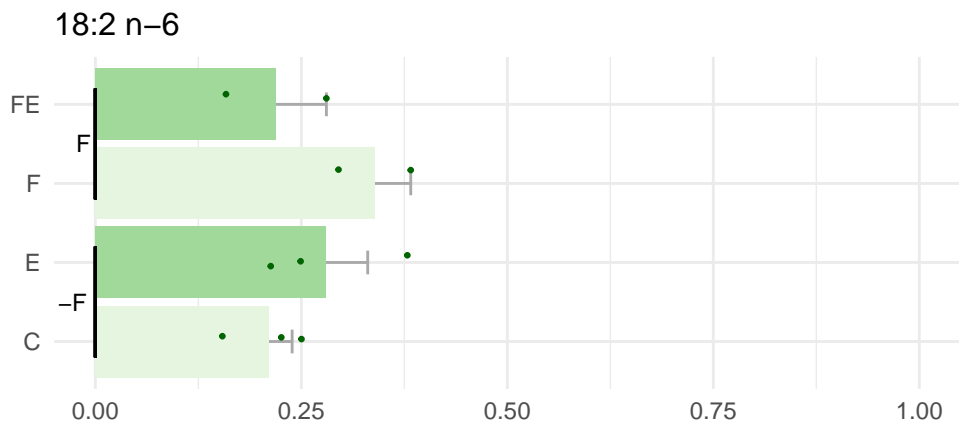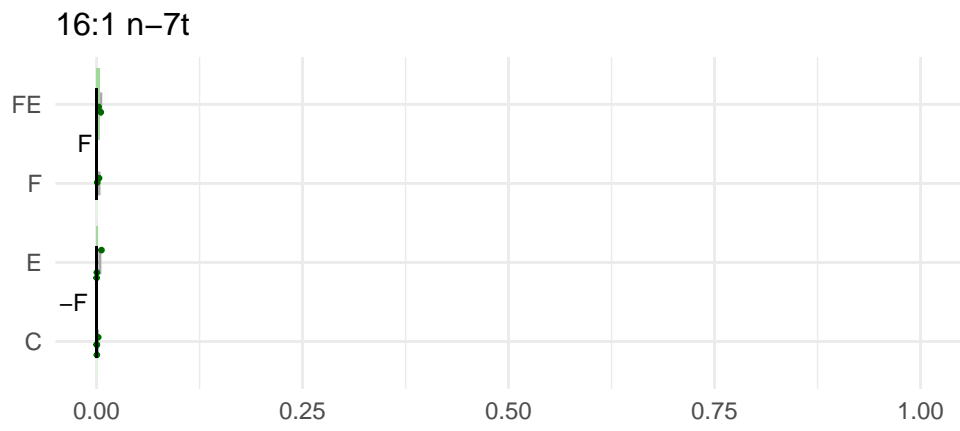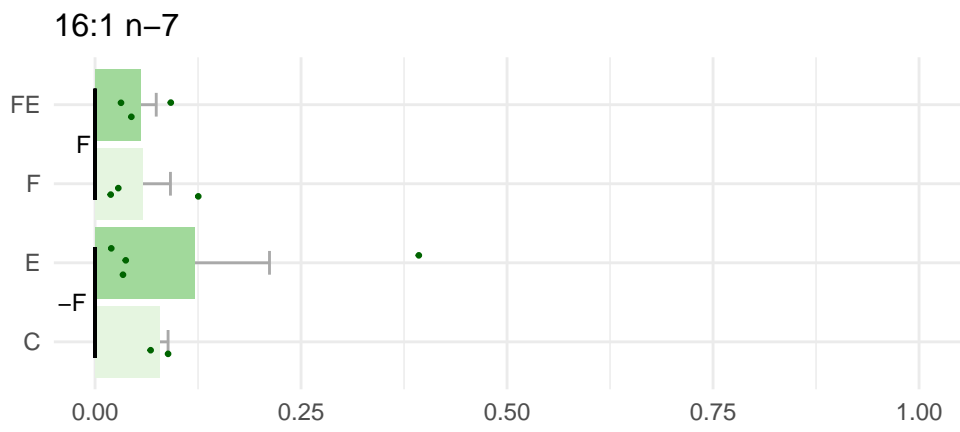

Supplement: Supplementary file 1 [file nutrients-15-03095-s001.zip › Figure S2.pdf]
